# Targeting interferon-stimulated gene of 20 kDa protein (*Isg20*) inhibits ribosome biogenesis to ameliorate the progression of renal fibrosis

**DOI:** 10.1371/journal.pone.0322639

**Published:** 2025-07-07

**Authors:** Xiaoming Liu, Huijuan Wang, Kai Wang, Ying Liu

**Affiliations:** 1 Department of Nephrology, Yantai Affiliated Hospital of Binzhou Medical University, Yantai, Shandong, P.R. China; 2 Department of Pathology, Yantaishan Hospital, Yantai, Shandong, P.R. China; National Institutes of Health, UNITED STATES OF AMERICA

## Abstract

Chronic kidney disease (CKD) is a global health issue that significantly threatens human health, with its incidence increasing annually. Renal fibrosis is characterized by the progressive loss of kidney function, leading to significant morbidity and mortality. Although ribosome biogenesis has been reported to be increased in several kidney diseases, its role in renal fibrosis remains unclear. This study investigates the role of the interferon-stimulated gene of 20 kDa protein (*Isg20*), an RNA exonuclease involved in several stages of ribosome biogenesis, in the progression of renal fibrosis. Bioinformatics analysis of Gene Expression Omnibus (GEO) datasets identified upregulation of ribosome biogenesis-related genes and *Isg20* expression in renal fibrosis samples. Using the unilateral ureteral obstruction (UUO)-induced renal fibrosis mouse model, we confirmed elevated *Isg20* expression, promoted renal fibrosis, and increased ribosome biogenesis. Knockdown of *Isg20* significantly reduced ribosome biogenesis, ameliorated kidney damage, inhibited pro-inflammatory cytokines levels and renal fibrotic changes, and decreased endoplasmic reticulum stress and cell apoptosis. Our findings suggest that *Isg20* exacerbates renal fibrosis by promoting ribosome biogenesis, ER stress and cell apoptosis highlighting a potential therapeutic target for renal fibrosis treatment.

## Introduction

Chronic kidney disease (CKD) is a global health issue that significantly threatens human health, with its incidence increasing annually [1–3]. Renal interstitial fibrosis (RIF) is generally considered the final common pathway leading to end-stage renal disease (ESRD) worldwide [4,5]. RIF is characterized by epithelial-mesenchymal transition (EMT) in renal tubular epithelial cells, extensive activation of fibroblasts, and massive deposition of extracellular matrix (ECM) [6,7], leading to renal scarring and promoting the progression to ESRD.

Ribosome biogenesis, during which the new ribosomes are generated, is fundamental for protein synthesis and cellular functions and processes, including cell proliferation, differentiation, apoptosis, development, and transformation [8]. Recent studies have highlighted its critical role in various diseases. For instance, increased ribosome biogenesis has been linked to the repair processes in myocardial ischemia/reperfusion injury [9], traumatic brain injury [10], and spinal cord injury [11]. In renal pathophysiology, ribosome biogenesis is crucial for cellular adaptation and response to injury [12]. Elevated ribosome biogenesis has been observed in clear cell renal cell carcinoma [13] and during kidney development [14], indicating its importance in both disease and normal physiological processes. Reportedly, transforming growth factor β1 (TGF-β1) could induce cell hypertrophy, regulate the mammalian target of rapamycin (mTOR) pathway, and mTORC1 could positively regulate ribosomal RNA transcription, ribosomal protein synthesis, and ribosome assembly [15–17]. Additionally, TGF-β1 has been found to induce the expression of ribosomal proteins in renal fibroblasts [18]. Given the essential role of the TGF-β signaling in renal fibrosis and CKD progression [19], the ribosome biogenesis pathway might be a potential target for anti-renal fibrosis.

Interferon-stimulated gene of 20 kDa protein (*ISG20*) is an RNA exonuclease known for its broad antiviral activity [20]. It is the human homolog of Rex4p, belonging to the Rex DEDD subfamily [21]. Rex proteins have been proven to participate in the processing of various rRNAs, including 5S and 5.8S [21]. Recent reports have shown that the homolog of *ISG20*, *ISG20L2*, is involved in the processing of 18S rRNA [22]. Furthermore, *ISG20* has been reported as a diagnostic biomarker for renal interstitial fibrosis, with elevated *ISG20* expression levels in RIF samples compared to normal samples; *ISG20* levels significantly increased in renal tubular epithelial cells stimulated by TGF-β [23]. Based on the critical roles of ribosome biogenesis and *ISG20* in various kidney-related conditions including renal fibrosis, we hypothesize that *ISG20* might facilitate renal fibrosis and CKD by affecting ribosome biogenesis.

To test this hypothesis, we conducted a series of experiments involving both bioinformatics analysis and *in vivo* validation using a mouse model of CKD. Differential gene expression analysis on Gene Expression Omnibus (GEO) datasets (GSE42303 and GSE121190) was conducted to identify genes associated with ribosome biogenesis in renal fibrosis samples. Ribosome biogenesis levels were confirmed in the unilateral ureteral obstruction (UUO)-induced renal fibrosis mouse model. *Isg20* expression was identified to be upregulated in datasets and validated in the mouse model. Finally, we investigated the effects of *Isg20* knockdown on renal fibrosis, ribosome biogenesis, endoplasmic reticulum (ER) stress, and apoptosis. Collectively, these comprehensive analyses aim to elucidate the role of *Isg20* in renal fibrosis and explore its potential as a therapeutic target.

## Materials and methods

### Bioinformatics analysis

Gene Expression Omnibus (GEO; https://www.ncbi.nlm.nih.gov/geo/) datasets GSE42303 (including 3 samples of UUO-induced mouse renal fibrosis samples and 3 controls) and GSE121190 (containing 3 UUO-induced renal fibrosis samples and 3 control samples) were downloaded and analyzed using limma package for differential gene expression. Metascape was used for functional enrichment analysis.

### Lentivirus package

The sequences for sh-NC (scramble sequence) and sh-*Isg20* are listed in Table 1. The forward and reverse shRNA oligos were annealed and ligated into the pLVX-shRNA1 vector. Then, the pLVX-shRNA1 vector was co-transfected with the packaging vectors into 293T cells. The culture supernatant was collected after 72h. The supernatant was filtered by a 0.45μm filter membrane into a speeding centrifuge tube. After 2 h of ultra-speed centrifuge (50000 g), the lentivirus (sh-*Isg20* and sh-NC) was collected and stored it in a −80°C until use.

**Table 1:** The sequence for qPCR primers and shRNA.

	Gene	Primer	Sequence (5’-3’)
qPCR primers	mmu-*5s rRNA*	Forward	TACGGCCATACCACCCTGAA
		Reverse	CTACAGCACCCGGTATTCCC
	mmu-*5.8s rRNA*	Forward	CTTAGCGGTGGATCACTCGG
		Reverse	GCAAGTGCGTTCGAAGTGTC
	mmu-*18s rRNA*	Forward	AGCTAATACATGCCGACGGG
		Reverse	TAGGGCAGACGTTCGAATGG
	mmu-*28s rRNA*	Forward	AGGGATAACTGGCTTGTGGC
		Reverse	TAAACCCAGCTCACGTTCCC
	mmu-*Rps19*	Forward	GAGGGGCTGAAAATGGTGGAA
		Reverse	ACCTGTCCAGCGATCCTGT
	mmu-*Isg20*	Forward	CCTGAAGGAGGATATGAGCAAGT
		Reverse	CCCGCCAGTTGTTCTGGAT
	mmu-*Rps9*	Forward	GGGAGCTGTTGACGCTAGAC
		Reverse	GCACCCCAATGCGAACAAG
	mmu-*Gapdh*	Forward	AGGTCGGTGTGAACGGATTTG
		Reverse	TGTAGACCATGTAGTTGAGGTCA
vector primer	(mmu) sh-NC	Forward	GATCCGCAGATGAAGGCACGGTCACGCTCGAGCGTGACCGTGCCTTCATCTGCTTTTTT
		Reverse	CTAGAAAAAAGCAGATGAAGGCACGGTCACGCTCGAGCGTGACCGTGCCTTCATCTGCG
	(mmu) sh-*Isg20*	Forward	GATCCGACCTGAAGCACGACTTCAATCTCGAGATTGAAGTCGTGCTTCAGGTCTTTTTG
		Reverse	AATTCAAAAAGACCTGAAGCACGACTTCAATCTCGAGATTGAAGTCGTGCTTCAGGTCG

### Animal model

Male C57BL/6 mice, aged 8–10 weeks and weighing between 20 and 22 grams, were sourced from the Hunan SJA Laboratory animal company (Changsha, China). They were kept in ventilated cages under a 12-hour light/12-hour dark cycle, with temperatures maintained at 21–23°C and humidity levels at 40%–60%. The mice had unlimited access to food and water. To create the UUO model, a flank incision was made to expose the left ureter, which was then tied at two points near the renal pelvis using 6–0 silk sutures. RNA interference treatment was initiated one day following the UUO procedure. For this, 1x10^7^ TU lv-*Isg20* (in 100 μl) was administered via tail-vein injection, on day 1 and day 7. The lv-sh-NC injection was set as a negative control. The UUO model was sustained for a duration of 14 days. All procedures were performed under anesthesia to minimize suffering. At the end of the procedure, all mice were anesthetized with 50 mg/kg sodium pentobarbital and sacrificed by cervical dislocation. The abdominal aorta blood collection was conducted, and serum was centrifuged to detect serum creatinine (SCr) and blood urea nitrogen (BUN). All the procedures were approved by the Laboratory Animal Welfare and Ethics Committee of Yantai Affiliated Hospital of Binzhou Medical University (No.2023−370).

### Serum biochemical analysis

The contents of SCr and BUN of mice in each experimental group were measured by commercial kits. The commercial kits, including SCr colorimetric assay kit (E-BC-K188-M) and BUN colorimetric assay kit (E-BC-K183-M), were commercial purchases from Elabscience (Wuhan, China). In brief, mice serum was collected using the same method described above. For SCr detection, enzyme solutions A and B were added to serum sample sequentially. The concentration of SCr of mice in each group was calculated after obtaining a standard curve. For BUN detection, an enzyme working solution, color developer, and alkaline sodium hypochlorite were added to serum sample sequentially. The absorbance at the optimal wavelength (515 nm for SCr and 580 nm for BUN) was measured using a microplate reader (Thermo Fisher Scientific, Waltham, USA). The concentrations of SCr and BUN in mice were determined by comparing the absorbance values to the standard curve.

### Histological analyses

The kidney tissue was fixed in paraffin and then sliced into slices that were 4 μm thick for hematoxylin and eosin (H&E) and Masson staining in histology. Tubular injury was identified in the renal tubules located in the cortical and outer stripes of the outer medulla. The injury was characterized by histological alterations including cell lysis, loss of brush boundary, and cast formation.

### qRT-PCR detecting ribosomal RNA

Total RNA was extracted from kidney tissues using the TRIzol reagent (Invitrogen, Carlsbad, USA) and the concentration and purity of the RNA were assessed using a NanoDrop spectrophotometer (Thermo Fisher Scientific). cDNA was synthesized from 1 μg of total RNA using the PrimeScript RT Reagent Kit (Takara, Tokyo, Japan) with random primers. Quantitative real-time PCR (qRT-PCR) was performed to detect the expression levels of ribosomal RNA (rRNA) species, including 5S, 5.8S, 18S, and 28S rRNA. Specific primers for each rRNA were designed and validated for efficiency and specificity. The qRT-PCR reactions were carried out using the SYBR Premix Ex Taq II (Takara) on a CFX96 Real-Time PCR Detection System (Bio-Rad, Hercules, USA). The relative expression levels of the rRNA species were calculated using the 2^-ΔΔCt^ method. The primers sequences are listed in Table 1.

### Immunoblotting

An assay kit for measuring bicinchoninic acid protein levels was used to extract protein samples from kidney samples (Thermo Fisher Scientific). In order to transfer 30−50 μg of protein onto a polyvinylidene difluoride membrane (Merck Millipore, Billerica, USA), it was separated by SDS-PAGE (Invitrogen). Primary antibodies against KIM-1 (30948–1-AP, Proteintech, Wuhan, China), NGAL (DF6816, Affinity Bioscience, Changzhou, China), E-cadherin (20874–1-AP, Protientech), α-SMA (14395–1-AP, Proteintech), FIBRONECTIN (15613–1-AP, Proteintech), COLLAGEN I (14695–1-AP, Proteintech), ISG20 (22097–1-AP, Proteintech), PERK (AF5304, Affinity Bioscience), IRE1 (DF7709, Affinity Bioscience), GRP78 (AF5366, Affinity Bioscience), BCL-2 (26593–1-AP, Proteintech), and BAX (50599–2-Ig, Proteintech) were used for incubating the membrane for one hour at room temperature after blocking with 5% BSA in tris-buffered saline-Tween 20. The membrane was then incubated overnight at 4°C. A secondary antibody (Abcam, Cambridge, USA) conjugated with HRP was incubated with the membrane for 90 minutes at room temperature after it had been washed with tris-buffered saline-Tween 20. The ECL Detection System was used to identify the signal. The full western blot images were listed as S1 File.

### Enzyme-linked immunosorbent assay (ELISA)

The levels of pro-inflammatory cytokines (TNF-α, IL-1β, and IL-6) in mouse renal tissues were measured using the commercial ELISA kits, as directed by the manufacturer. ELISA kits for TNF-α (CSB-E04741m), IL-1β (CSB-E08054m), and IL-6 (CSB-E04639m) were procured from CUSABIO (Wuhan, China).

### Immunohistochemistry staining (IHC staining)

The UltraSensitive SP IHC Kit (Maixin, Fuzhou, China) was used for IHC staining. After formalin-fixed and paraffin-embedded kidney sections were deparaffinized and rehydrated, antigen retrieval was accomplished by boiling the sections in an EDTA solution for 10 minutes. Sections were treated with 3% hydrogen peroxide to suppress endogenous peroxidase activity. After the primary antibody against ISG20 was incubated with the sections overnight at 4°C, a biotinylated secondary antibody, and a streptavidin peroxidase solution were added. Chromogen diaminobenzidine was utilized to final stain the section for 5–10 min.

### TUNEL staining

Cell apoptosis was identified using the colorimetric TUNEL apoptosis assay kit (Beyotime, Shanghai, China) following the manufacturer’s instructions. The number of TUNEL-positive cells (brown color) was determined in the outer medulla and kidney cortex regions of each specimen.

### Statistical analysis

Each animal experiment was repeated at least six times (biological repetition). The statistical analysis was performed using the SPSS 21.0 (IBM, Armonk, NY, USA), and the data were reported as the mean ± standard deviation (SD). Kolmogorov–Smirnov test showed whether the data were in normal distribution. The statistical significance was established by doing a Student’s *t*-test between two groups or a one-way ANOVA followed by the LSD test or Dunnett T3 test in groups consisting of more than two. The Kruskal-Wallis was used for non-parametric statistical analysis. A *P* value less than 0.05 was deemed to be statistically significant. The statistical analysis results were reported in S2 File.

## Results

### Bioinformatics analysis identifies differentially expressed genes (DEGs) associated with ribosome biogenesis in renal fibrosis

**Fig 1:**
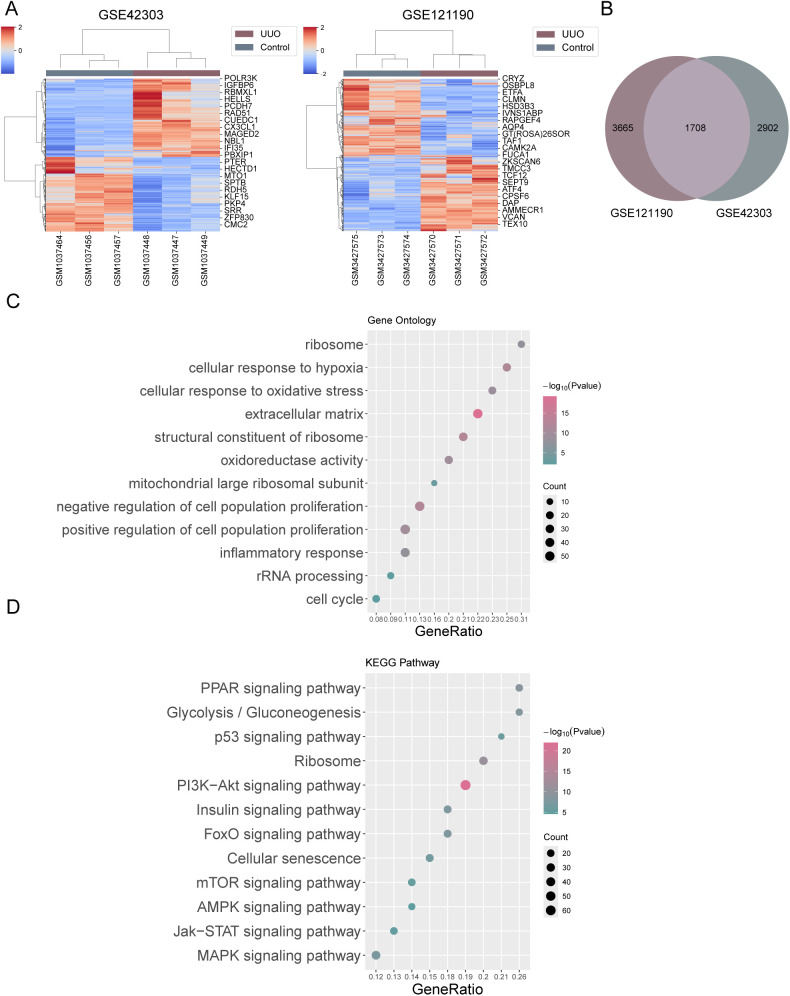
Bioinformatics analysis identifies differentially expressed genes associated with ribosome biogenesis in renal fibrosis. (A) Differential gene expression analysis was performed on GEO datasets GSE42303 (including 3 samples of UUO-induced renal fibrosis samples and 3 controls) and GSE121190 (containing 3 UUO-induced renal fibrosis samples and 3 control samples) using the limma package in R studio. (B) Venn diagram showing overlapping differentially expressed genes with consistent expression trends in both datasets. (C-D) Metascape was used for the Gene Ontology (GO) functional and Kyoto Encyclopedia of Genes and Genomes (KEGG) signaling pathway enrichment annotation of the differentially expressed genes.

Differential gene expression analysis was performed on GEO datasets GSE42303 (including 3 samples of mouse UUO-induced renal fibrosis samples and 3 controls) and GSE121190 (containing 3 UUO-induced mouse renal fibrosis samples and 3 control samples) using the limma package in R studio. The analysis identified 4610 and 5373 differentially expressed genes, respectively (|logFC| > 0.5, P.Value<0.05; Fig 1A). Next, DEGs identified from the two datasets were compared and a total of 1708 overlapping DEGs with consistent expression trends were found (Fig 1B). Metascape was used for the Gene Ontology (GO) functional enrichment annotation of the 1708 DEGs; the analysis highlighted their association with various biological processes, including extracellular matrix, structural components of ribosomes, negative regulation of cell population proliferation, cellular response to hypoxia, positive regulation of cell population proliferation, oxidoreductase activity, cellular response to oxidative stress, inflammatory response, ribosomes, mitochondrial large ribosomal subunits, rRNA processing, cell cycle, etc. (Fig 1C). KEGG Pathway analysis showed that these DEGs were significantly associated with the PI3K-Akt signaling pathway, ribosomes, the PPAR signaling pathway, glycolysis/glycolysis signaling pathway, FoxO signaling pathway, insulin signaling pathway, MAPK signaling pathway, cellular senescence, the p53 signaling pathway, the mTOR signaling pathway, the Jak-STAT signaling pathway, and the AMPK signaling pathway (Fig 1D). GO and KEGG analysis showed that these genes might affect renal fibrosis by influencing ribosome biogenesis.

### Elevated ribosome biogenesis in a UUO-induced renal fibrosis mouse model

**Fig 2:**
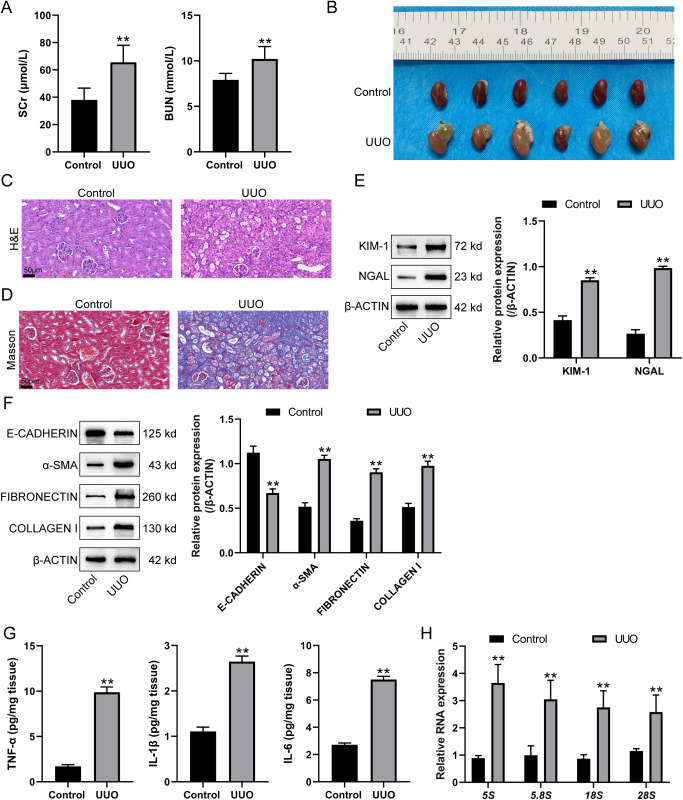
Elevated ribosome biogenesis in a unilateral ureteral obstruction (UUO)-induced renal fibrosis mouse model. Mice were randomly allocated into UUO and control groups. UUO was conducted as described. (A) Serum creatinine (SCr) and blood urea nitrogen (BUN) levels were measured from serum samples. (B) Gross morphology of kidneys. (C) Hematoxylin and eosin (H&E) staining was performed to assess pathological damage in the renal cortex. (D) MASSON staining was performed to detect collagen production in the renal cortex. (E) Immunoblotting was applied to assess the expression levels of KIM-1 and NGAL, both markers of tubular injury, in kidney tissues. (G) The levels of TNF-α, IL-1β, and IL-6 in mouse renal tissues were measured by ELISA. (F) Immunoblotting was performed to detect the protein levels of E-CADHERIN, α-SMA, FIBRONECTIN, and COLLAGEN I. (H) qRT-PCR was performed to measure rRNA levels in the kidneys. Data are analyzed using unpaired Student’s t-test and presented as the mean ± SD. N = 6 (biological replicates) for A, C, D, G and H. N = 3 (biological replicates) for E and F. ** P < 0.01 compared to control group.

For validating the findings by bioinformatics analysis, a UUO-induced renal fibrosis model was established in mice. Mice were randomly allocated into UUO and control groups. UUO was conducted as described. For model verification, serum creatinine (SCr) and blood urea nitrogen (BUN) levels were measured from serum samples obtained via abdominal aorta blood collection and centrifugation. Fig 2A shows that UUO operation significantly elevated the levels of SCr and BUN compared to the control group. Gross morphology of kidneys was shown in Fig 2B. Compared to the control group, the ureteric blockage causes a significant alteration in the appearance of the kidney, resulting in a whiter and more taut texture that could be felt upon touch (Fig 2B). H&E staining was performed to assess pathological damage in the renal cortex. The normal, unaltered native kidney shows glomeruli encircled by the dense tubules of the tubulointerstitium, with many tubules displaying a visible lumen (Fig 2C). After the obstruction of the ureter, an enlargement and expansion of the ureter and renal pelvis was observed, which was accompanied by a progressive enlargement of the renal tubules (Fig 2C). Consistently, MASSON staining revealed higher collagen production in the renal cortex of UUO-induced mice (Fig 2D). To assess renal damage, the expression of KIM-1 and NGAL, which are markers of tubular injury, was quantified in the kidney tissues of mice. The KIM-1 and NGAL expressions were significantly elevated in the UUO group compared to the control group (Fig 2E). The fibrotic alterations were confirmed by the levels of fibrotic markers; Fig 2F shows that the level of E-CADHERIN was significantly decreased, whereas α-SMA, FIBRONECTIN, and COLLAGEN I were remarkably increased in UUO mice kidney compared to the control group. The expression levels of pro-inflammatory cytokines (TNF-α, IL-1β, and IL-6) in mouse renal tissues were measured by ELISA. As shown in Fig 2G, in the UUO group, there was a marked increase in the levels of TNF-α, IL-1β, and IL-6 compared to the control group. The rRNA levels in the kidneys were examined to evaluate the ribosome biogenesis; Fig 2H shows that *5s*, *5.8s*, *18s*, and *28s* rRNA levels were drastically elevated in UUO mice kidneys compared to the control group. These data suggest that the ribosome biogenesis is enhanced in the UUO-induced renal fibrosis mouse model.

### Renal fibrosis is accompanied by significant upregulation of *Isg20* and ribosome biogenesis increase

**Fig 3:**
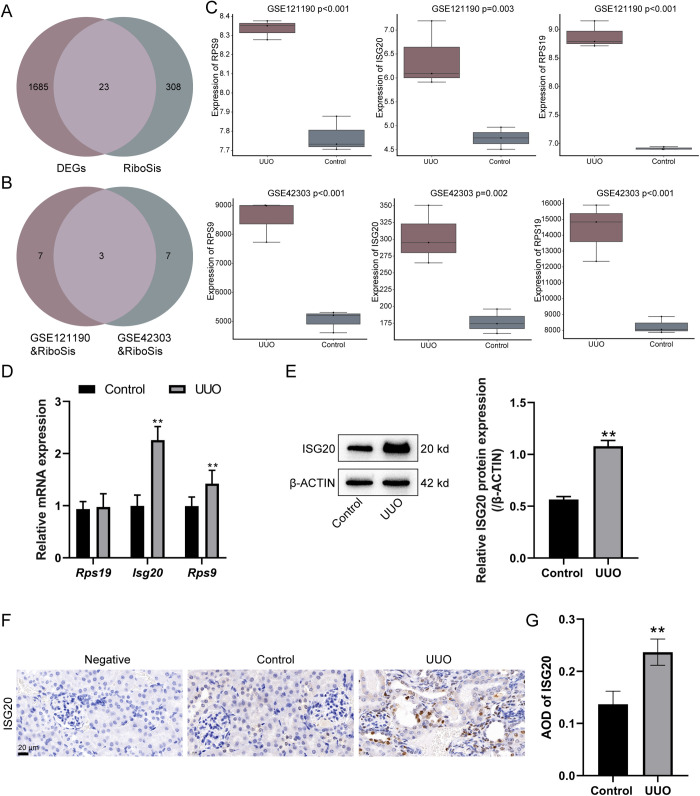
Renal injury is accompanied by significant upregulation of *Isg20* and ribosome biogenesis increase. (A) Intersection analysis identifying 1708 overlapping differentially expressed genes related to 331 ribosome biogenesis and 23 overlapping genes were identified. (B) Among these 23 overlapping genes, DEGs with the top 10 log_2_|FC| values were compared and 3 were overlapping. (C) Expression levels of *Rps19*, *Rps9*, and *Isg20* in renal fibrosis samples from datasets GSE42303 and GSE121190. (D) The mRNA expression levels of *Rps19*, *Rps9*, and *Isg20* were determined in mice kidneys using qRT-PCR. N = 6 (biological replicates). (E) ISG20 protein level was determined in mice kidneys using Immunoblotting. N = 3 (biological replicates). (F) IHC quantitative analysis was performed to evaluate the cytoplasm and nuclei distribution of ISG20. N = 3 (biological replicates). Data are analyzed using unpaired Student’s t-test and presented as the mean ± SD. ** P < 0.01 compared to control group.

According to a previous study [24], 331 ribosome biogenesis-related genes (RiboSis) were identified; by intersections with DEGs identified from GSE42303 and GSE121190, 23 ribosome biogenesis-related DEGs were obtained (Fig 3A). Of the 23 ribosome biogenesis-related DEGs, the top 10 genes with log_2_|FC| value in GSE42303 and GSE121190 were analyzed and *Rps19*, *Isg20*, and *Rps9* were shown to be top10 ribosome biogenesis-related factors in the two datasets (Fig 3B). The expression levels of *Rps19*, *Isg20*, and *Rps9* were significantly upregulated in renal fibrosis samples from both the GSE42303 and GSE121190 datasets (Fig 3C, S3 File).

For verifying the correlation of renal injury, ribosome biogenesis, and ISG20 upregulation, the mRNA levels of identified DEGs *Rps19*, *Isg20*, and *Rps9* were determined in mice kidneys using qRT-PCR. Fig 3D shows that *Isg20* and *Rps9* were significantly elevated in UUO-induced mice kidneys, with *Isg20* being the more upregulated. Consistently, ISG20 protein levels were significantly elevated in UUO-induced mice kidneys compared to the control group (Fig 3E). ISG20 abundance in UUO-induced mice kidneys was confirmed by IHC staining as well (Fig 3F). IHC quantitative analysis was performed to evaluate the cytoplasm and nuclei distribution of ISG20. Fig 3G shows that the ISG20 protein levels were significantly elevated in the UUO mice kidneys compared to the control group. These data suggest that renal fibrosis is accompanied by significant upregulation of *Isg20* and ribosome biogenesis increase.

### Knockdown of *Isg20* ameliorates renal fibrosis and reduces ribosome biogenesis

**Fig 4:**
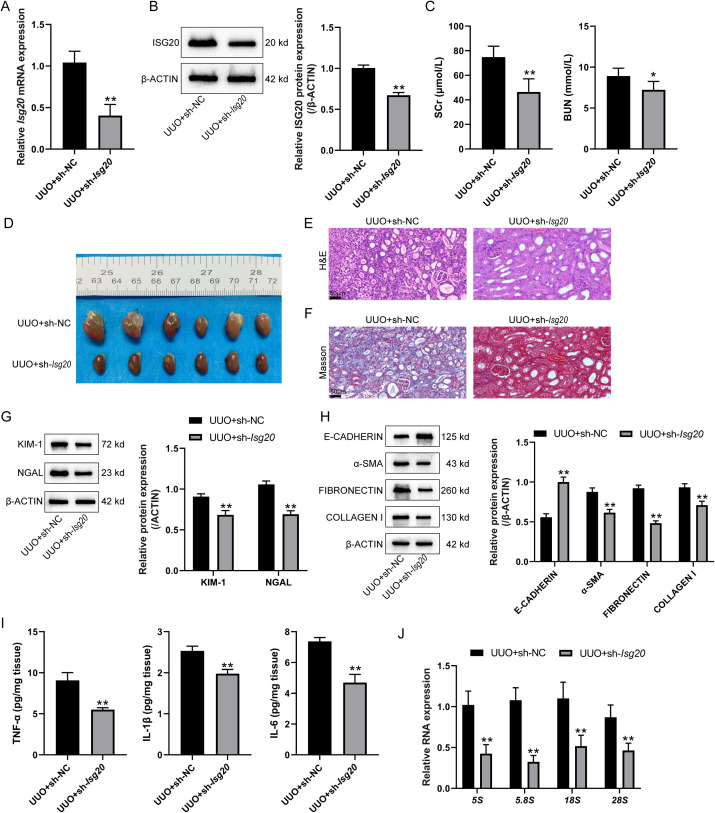
Knockdown of *Isg20* ameliorates renal fibrosis and reduces ribosome biogenesis. *Isg20* knockdown was achieved in UUO-induced CKD mice models by introducing short hairpin RNA against *Isg20*. (A-B) *Isg20* knockdown was confirmed in mice kidneys using qRT-PCR and Immunoblotting. (C) SCr and BUN levels measured from serum samples obtained via abdominal aorta blood collection and centrifugation. (D) Gross morphology of kidneys. (E) H&E staining was performed to assess pathological damage in the renal cortex. (F) MASSON staining was performed to detect collagen production in the renal cortex. (G) Immunoblotting was used to determine the protein levels of KIM-1 and NGAL in kidney tissues. (H) Immunoblotting was performed to detect the protein levels of E-CADHERIN, α-SMA, FIBRONECTIN, and COLLAGEN I in kidney tissues. (I) The expression levels of TNF-α, IL-1β, and IL-6 in mice renal tissues were measured by ELISA. (J) qRT-PCR was performed to detect rRNA levels in kidney tissues. Data are analyzed using unpaired Student’s t-test and presented as the mean ± SD. N = 6 (biological replicates) for A, C, D, E, I and J. N = 3 (biological replicates) for B, G and H. ** P < 0.01 compared to UUO + sh-NC group.

For the specific role of *Isg20* in renal fibrosis and ribosome biogenesis, *Isg20* was knocked down in the UUO-induced renal fibrosis model in mice and confirmed using qRT-PCR and Immunoblotting (Fig 4A and 4B). Regarding renal injury, Fig 4C shows that knockdown of *Isg20* significantly reduced the levels of SCr and BUN compared to the UUO + sh-NC group. The gross morphology of kidneys is shown in Fig 4D. Compared to the UUO + sh-NC group, knockdown of *Isg20* resulted in a noticeable improvement in the appearance of the kidney, with a less taut and more normal texture (Fig 4D). H&E staining was performed to assess pathological damage in the renal cortex. In the UUO + sh-NC group, the kidney shows severe damage with enlarged and dilated renal tubules, whereas knockdown of *Isg20* mitigated these pathological changes (Fig 4E). Consistently, MASSON staining revealed lower collagen production in the renal cortex of UUO + sh-*Isg20* mice compared to the UUO + sh-NC group (Fig 4F). Silencing of *Isg20* resulted in a decrease in KIM-1 and NGAL expression compared to the sh-NC group (Fig 4G). Fig 4H shows that *Isg20* knockdown significantly increased E-CADHERIN level and decreased α-SMA, FIBRONECTIN, and COLLAGEN I protein levels compared to the UUO + sh-NC group. Furthermore, as demonstrated in Fig 4I, silencing of *Isg20* in UUO mice led to a reduction in the expression of TNF-α, IL-1β, and IL-6. The rRNA levels in the kidneys were examined to evaluate ribosome biogenesis. Fig 4J shows that *5s*, *5.8s*, *18s*, and *28s* rRNA levels were significantly reduced in the UUO + sh-*Isg20* group compared to the UUO + sh-NC group, suggesting suppressed ribosome biogenesis. These data suggest that knockdown of *Isg20* ameliorates renal fibrosis and reduces ribosome biogenesis in the UUO-induced renal fibrosis mouse model.

### Knockdown of *Isg20* potentially ameliorates endoplasmic reticulum (ER) stress and cell death by inhibiting ribosome biogenesis

**Fig 5:**
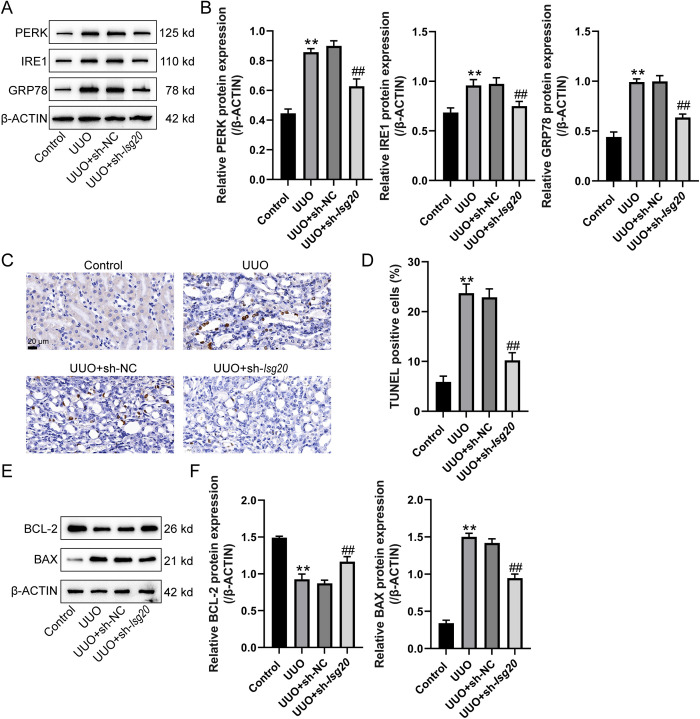
Knockdown of *Isg20* potentially ameliorates endoplasmic reticulum (ER) stress and cell death by inhibiting ribosome biogenesis. Mice were randomly allocated into four groups: Control group, UUO group, UUO + sh-NC group, and UUO + sh-*Isg20* group and received corresponding operation and treatments. (A-B) The protein levels of PERK, IRE1, and GRP78 in kidney tissues were determined using Immunoblotting. N = 3 (biological replicates). (C) TUNEL staining was performed to detect cell apoptosis in the renal cortex of mice. N = 6 (biological replicates). (D) Quantification of apoptosis rate in kidney tissues from TUNEL staining. (E-F) The protein levels of apoptosis-related factors BCL-2 and BAX in kidney tissues were determined using Immunoblotting. N = 3 (biological replicates). Data are analyzed using one-way ANOVA followed LSD test for (B, D and F). ** P < 0.01 compared to control group; ## P < 0.01 compared to UUO + sh-NC group.

Severe ER stress is associated with the development of degenerative and fibrotic disorders [25]. To investigate the potential mechanisms by which *Isg20* knockdown ameliorates renal fibrosis, we assessed ER stress and cell death in a UUO-induced renal fibrosis model. Mice were randomly allocated into four groups: Control group, UUO group, UUO + sh-NC group, and UUO + sh-*Isg20* group, and received corresponding operations and treatments. The protein levels of PERK, IRE1, and GRP78 in kidney tissues were determined using Immunoblotting. As shown in Fig 5A and 5B, ER stress markers were significantly upregulated in UUO-induced renal fibrosis mice, whereas the knockdown of *Isg20* significantly reduced the expression of these ER stress markers compared to the UUO and UUO + sh-NC groups. TUNEL staining was performed to detect cell apoptosis in the renal cortex of mice. The results in Fig 5C and 5D demonstrate that cell apoptosis was drastically facilitated in UUO-induced mice, whereas the knockdown of *Isg20* markedly decreased the apoptosis rate in kidney tissues compared to the UUO and UUO + sh-NC groups. Additionally, the protein levels of apoptosis-related factors BCL-2 and BAX in kidney tissues were determined using Immunoblotting. Fig 5E and 5F show that in UUO and sh-NC-injected mice, BCL-2 was decreased but BAX was increased compared to the control group; *Isg20* knockdown resulted in increased BCL-2 expression and decreased Bax expression compared to the UUO and UUO + sh-NC groups, indicating reduced apoptosis. These findings suggest that *Isg20* knockdown potentially ameliorates ER stress and cell death by inhibiting ribosome biogenesis in the UUO-induced renal fibrosis model.

## Discussion

In this study, 1708 DEGs in renal fibrosis were identified based on GSE42303 and GSE121190 datasets. GO functional and KEGG signaling pathway enrichment annotation of these 1708 DEGs highlighted their association with ribosome. Further analysis yielded three ribosome biogenesis-related DEGs, including *Rps19*, *Rps9*, and *Isg20*, which were significantly upregulated in renal fibrosis samples. Validation of these findings in a UUO-induced renal fibrosis mouse model showed that UUO significantly elevated SCr and BUN levels, led to histopathological alterations and renal damage, decreased E-CADHERIN and increased α-SMA, FIBRONECTIN, and COLLAGEN I, and upregulated pro-inflammatory cytokines (TNF-α, IL-1β, and IL-6) and rRNA levels, indicating the occurrence of renal injuries accompanied by enhanced ribosome biogenesis. *Isg20* upregulation was confirmed, correlating with increased ribosome biogenesis. Knockdown of *Isg20* in the UUO model significantly reduced SCr and BUN levels, improved kidney morphology, decreased pathological damage and renal damage, increased E-CADHERIN and decreased α-SMA, FIBRONECTIN, and COLLAGEN I, and suppressed pro-inflammatory cytokines and rRNA levels. Furthermore, *Isg20* knockdown ameliorated ER stress and cell death, as evidenced by reduced levels of ER stress markers, decreased apoptosis rates, and altered expression of apoptosis-related proteins BCL-2 and BAX. These findings suggest that *Isg20* plays a critical role in the progression of renal fibrosis through ribosome biogenesis, ER stress, and apoptosis regulation.

The different roles of ribosome biogenesis in kidney diseases have been reported previously. In the case of diabetic nephropathy, it has been discovered that the mTOR/p70 ribosomal S6 kinase/4E-binding protein 1 (mTOR/P70S6K/4EBP1) pathway promotes the production of ribosomes and protein synthesis after the activation of mTOR complex 1. Berberine, on the other hand, could decrease podocyte apoptosis induced by high glucose by regulating this pathway [26]. In wild-type male mice, ribosome biogenesis is linked to increased albuminuria, urinary kidney injury molecule-1, hypertension, kidney p70S6K phosphorylation, and kidney matrix accumulation under diabetic conditions, suggesting common pathways in renal disorders [27]. By using ribosome profiling, Yang et al. [28] recognized secreted micropeptide *C4f48* as a candidate molecule involved in renal fibrosis. The levels of *C4f48* RNA and protein were increased in tubular epithelial cells in both human and experimental CKD. Elevated levels of serum CF48 were seen in individuals with CKD and were found to be associated with declining kidney function, advancing CKD stage, and the extent of active interstitial fibrosis [28]. In this study, bioinformatics analysis showed that renal fibrosis was accompanied by a sharp increase in ribosome production. In a UUO-induced renal fibrosis model in mice, enhanced ribosome biogenesis was accompanied by a decreased level of epithelial marker E-CADHERIN [29] and increased fibrotic markers α-SMA, FIBRONECTIN, and COLLAGEN I [30,31], further suggesting the role of ribosome biogenesis in renal fibrosis and CKD.

To identify key factors regulating ribosome biogenesis in renal fibrosis, we analyzed DEGs related to ribosome biogenesis in renal fibrosis samples. Among these, *Rps19*, *Rps9*, and *Isg20* were significantly upregulated in UUO-induced renal fibrosis mouse kidney samples, with *Isg20* showing the highest level of upregulation. The elevated levels of rRNA in renal fibrosis mice models further suggest a role for *Isg20* in ribosome biogenesis under renal fibrosis conditions. *Rps19*, a constituent of the 40S small ribosomal subunit, has been discovered to interact with the pro-inflammatory cytokine macrophage MIF, hence acting as a new therapeutic agent for inflammatory kidney disease [32]. Polystyrene (PS)-microplastics (MPs) have been discovered to induce many harmful consequences, including mitochondrial damage, stimulation of inflammation response in kidney cells, and accumulation in the kidney tissues of mice [33]. A study was conducted to discover stable reference genes for gene expression analysis in kidney samples with MP bioaccumulation, and *Rps9* was identified as one of the most stable reference genes [34]. *ISG20* has also been recognized as one of the diagnostic gene biomarkers associated with immune infiltration in patients with renal fibrosis by a machine learning-based analysis; *ISG20* siRNA significantly suppressed the progression of renal fibrosis in vitro [35]. Among the three identified DEGs, *Isg20* was found to be the more upregulated in a UUO-induced renal fibrosis mice model. In addition, *Isg20* was predominantly located in the nucleus, further suggesting its potential role in ribosome biogenesis under renal fibrosis conditions.

Regarding the specific effects and mechanisms of *Isg20* in renal fibrosis, the knockdown of *Isg20* was achieved in the renal fibrosis mice models. In the renal fibrosis model, *Isg20* knockdown significantly ameliorated kidney injury, as evidenced by improved kidney function and morphology. The reduction in serum creatinine and blood urea nitrogen levels [36,37], alongside a noticeable improvement in kidney appearance, underscores the potential therapeutic benefits of targeting *Isg20*. The histological analysis further confirmed that *Isg20* knockdown mitigates severe renal damage, as shown by reduced tubular dilation and collagen production [38]. Additionally, the observed decrease in rRNA levels in the kidneys indicates that *Isg20* knockdown effectively suppresses ribosome biogenesis. Previously, *ISG20* has been reported to act as a mediator of CX3CL1 production, thereby inducing glomerular inflammation, particularly in patients with lupus nephritis [39]. ER stress related to both genetic and environmental factors has been identified in CKD. ER stress could induce epithelial cell apoptosis, and promote kidney fibroblast differentiation and ECM deposition, promoting profibrotic cytokine production from epithelial and immune cells, finally resulting in renal fibrosis [25]. It has been reported that suppression of ribosome synthesis attenuated ER stress and reduced cell death in I/R tissues [40]. Whlie in gastric cancer, ER stress, and impaired ribosome synthesis ultimately induced apoptotic cell death [41]. Therefore, the relationship between ribosome synthesis and ER stress exhibited tissue and disease specificity. Here, *Isg20* knockdown-caused ribosome biogenesis suppression likely contributes to the alleviation of ER stress and a reduction in cell apoptosis. The modulation of apoptosis-related proteins BCL-2 and BAX further supports the protective role of *Isg20* knockdown in reducing cell death. Given the reported roles of *Isg20* in lupus nephritis [39] and clear-cell renal-cell carcinoma [42], our results highlight the pivotal and multifaceted roles of *Isg20* in renal disorders; furthermore, *Isg20* likely facilitates ribosome biogenesis to promote renal fibrosis progression.

However, it is important to acknowledge the limitations that still exist within this study. One of the primary limitations of this study is the lack of direct evidence linking the observed protection to inhibition of ribosome biogenesis. Although ribosome biogenesis was assessed through qPCR analysis, further experimental validation is required to conclusively establish whether alterations in ribosome activity are the driving factor behind the protective effects of ISG20 knockdown. Additionally, while the role of *Isg20* in RNA oxidation [43] and immune response regulation [44] has been well documented, the potential involvement of these alternative mechanisms in fibrosis protection was not directly explored in this study. Further investigation into how *Isg20* modulates these pathways could provide a more comprehensive understanding of its multifaceted role in kidney fibrosis.

In conclusion, this study demonstrates that *Isg20*, upregulated in renal fibrosis mice kidney, may play a critical role in the progression of renal fibrosis through its regulation of ribosome biogenesis, ER stress, and apoptosis. Targeting *Isg20* may provide a potential therapeutic strategy for ameliorating kidney injury by reducing ribosome biogenesis, alleviating ER stress, and decreasing cell death.

## Supporting information

S1 File Original three repeated western blot images. (PDF)

S2 File Statistical analysis results report. (PDF)

S3 File Expression of RiboSis-related differentially expressed genes with the top 10 log_2_|FC| values from GSE42303 and GSE121190. (DOCX)
