# Supplementary material for: Targeting interferon-stimulated gene of 20 kDa protein (Isg20) inhibits ribosome biogenesis to ameliorate the progression of renal fibrosis
Source: PLoS One. 2025 Jul 7;20(7):e0322639. doi: 10.1371/journal.pone.0322639 (PMC12233288; doi:10.1371/journal.pone.0322639)
Supplement: S1 File — (PDF) [file pone.0322639.s001.pdf]

Original whole membrane of western blots in Fig2E

repeat_1: (Used in the manuscript)

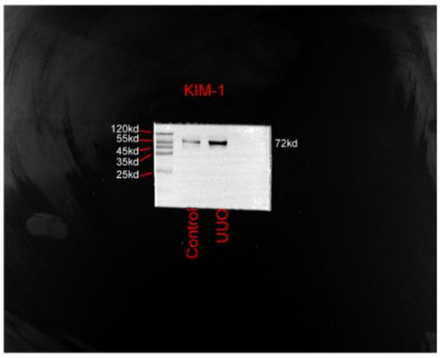

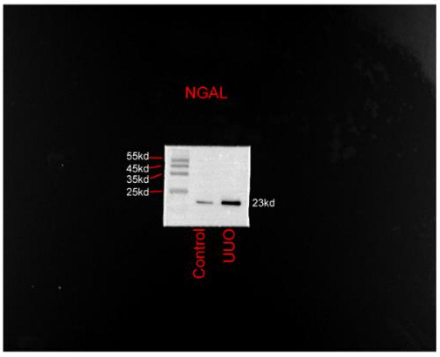

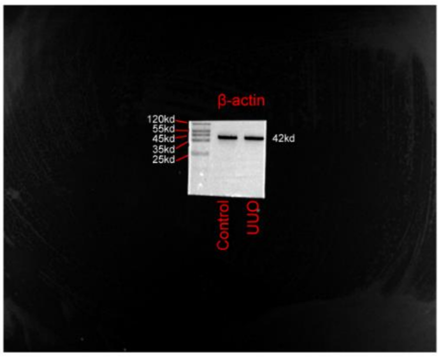

repeat_2

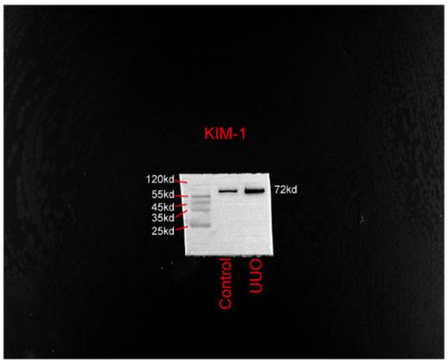

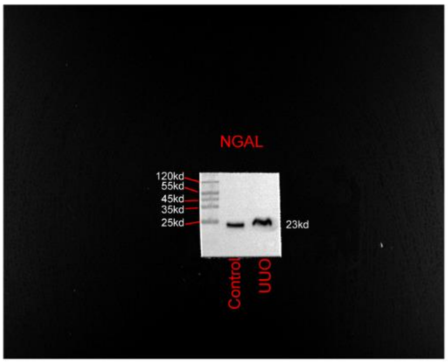

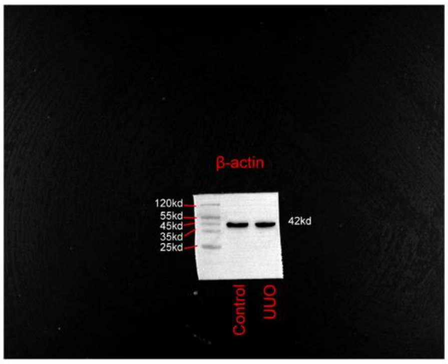

repeat_3

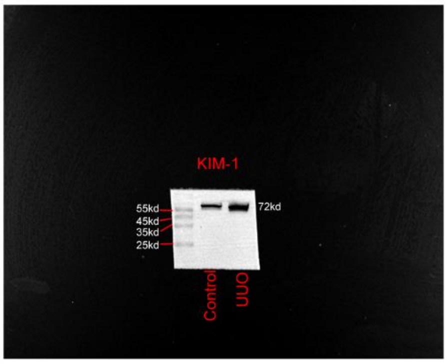
KIM-1
55kd
45kd
35kd
25kd
72kd
Control
UUO

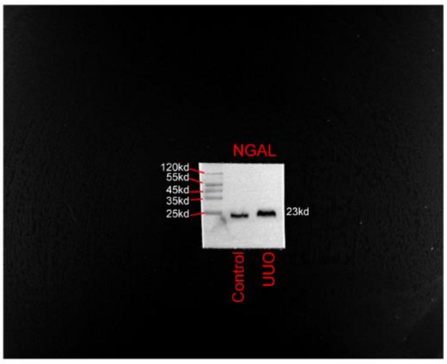
NGAL
120kd
55kd
45kd
35kd
25kd
23kd
Control
UUO

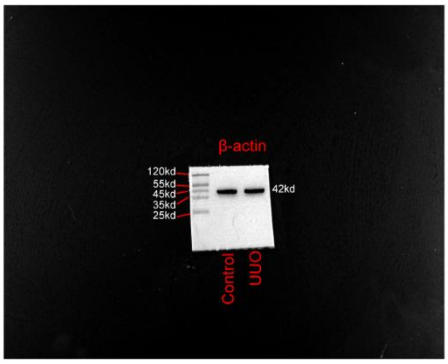
β-actin
120kd
55kd
45kd
35kd
25kd
42kd
Control
UUO

Original whole membrane of western blots in Fig2F

repeat_1: (Used in the manuscript)

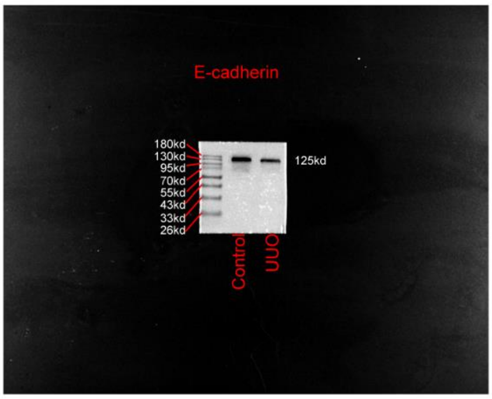

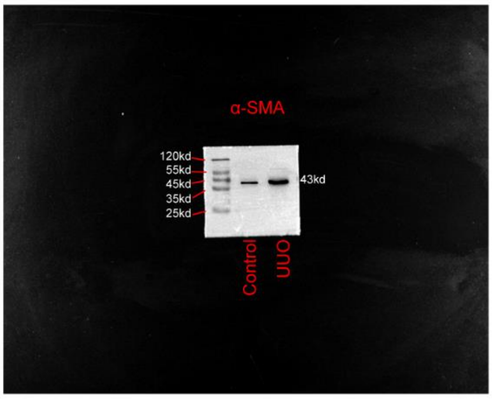

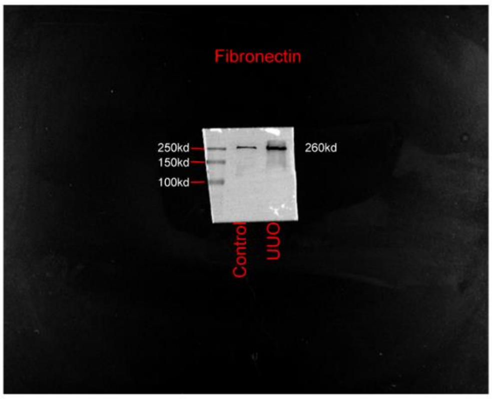

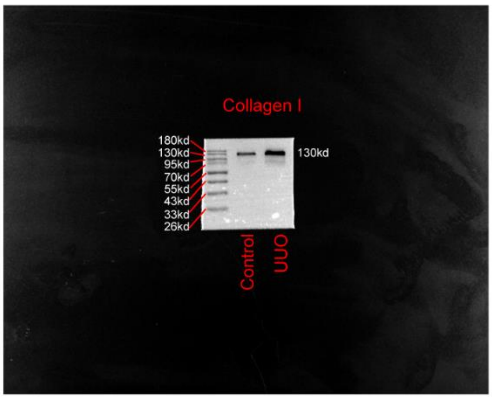

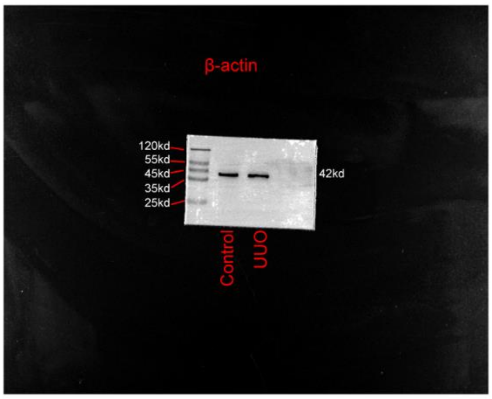

repeat_2

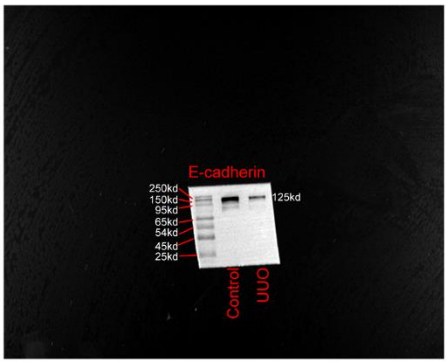
E-cadherin
250kd
150kd
95kd
65kd
54kd
45kd
25kd
125kd
Control
UUO

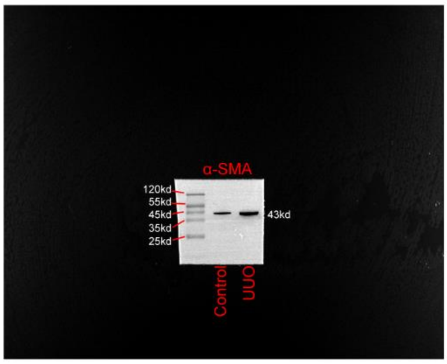
α-SMA
120kd
55kd
45kd
35kd
25kd
43kd
Control
UUO

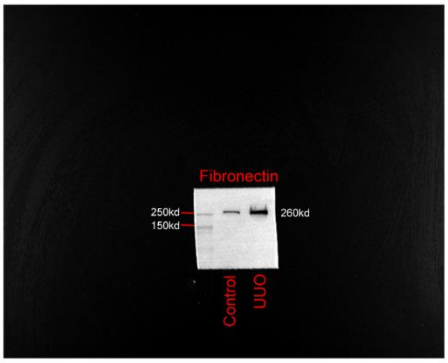
Fibronectin
250kd
150kd
260kd
Control
UUO

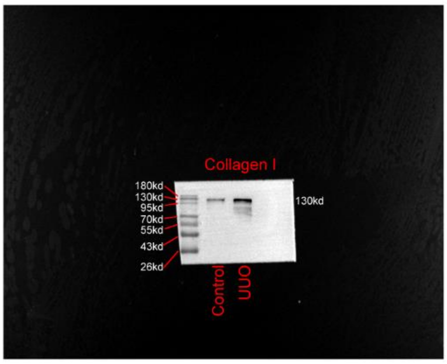
Collagen I
180kd
130kd
95kd
70kd
55kd
43kd
26kd
130kd
Control
UUO

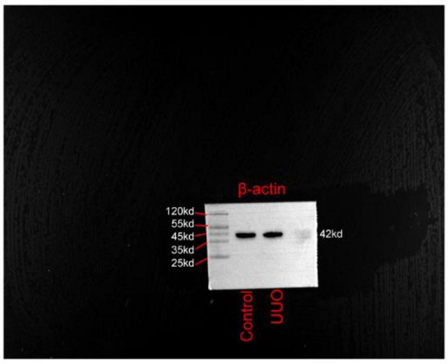
β-actin
120kd
55kd
45kd
35kd
25kd
42kd
Control
UUO

repeat_3

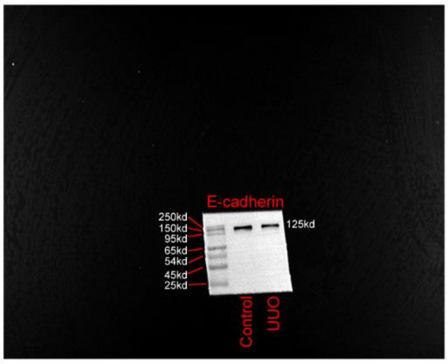
E-cadherin
250kd
150kd
95kd
65kd
54kd
45kd
25kd
125kd
Control
UUO

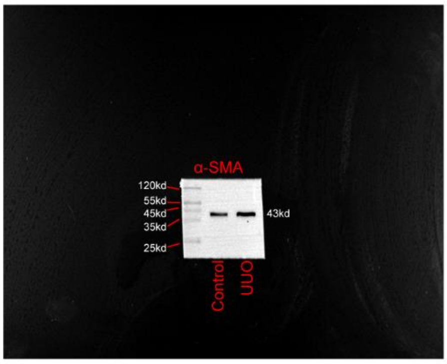
α-SMA
120kd
55kd
45kd
35kd
25kd
43kd
Control
UUO

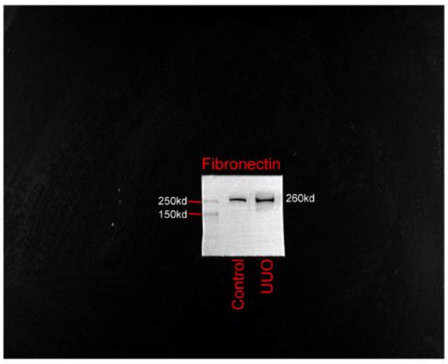
Fibronectin
250kd
150kd
260kd
Control
UUO

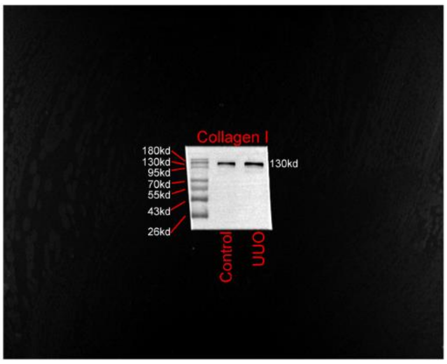
Collagen I
180kd
130kd
95kd
70kd
55kd
43kd
26kd
130kd
Control
UUO

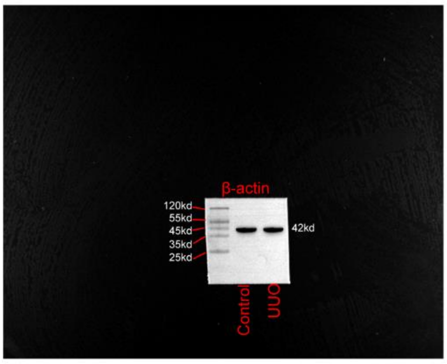
β-actin
120kd
55kd
45kd
35kd
25kd
42kd
Control
UUO

Original whole membrane of western blots in Fig3E

repeat_1: (Used in the manuscript)

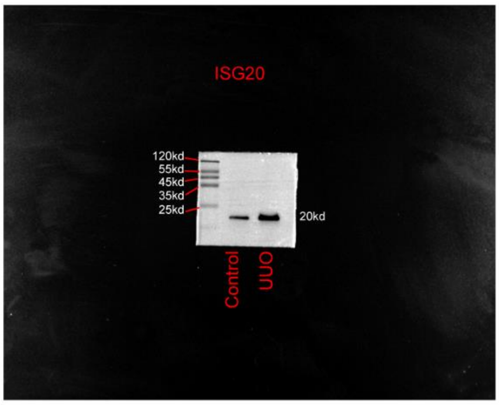

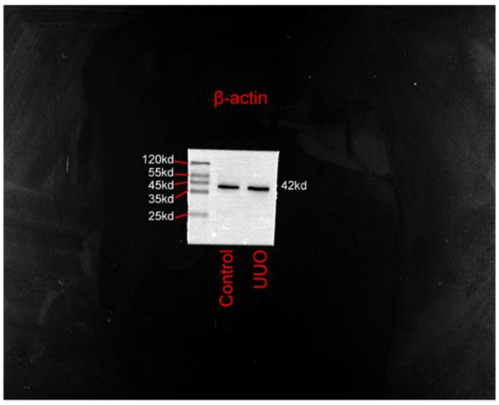

repeat_2

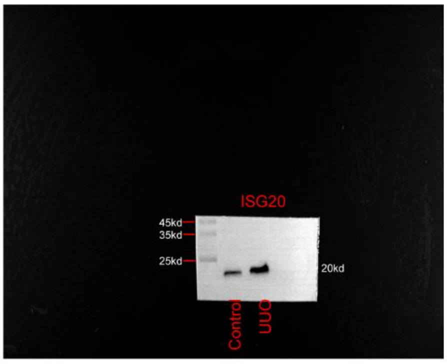

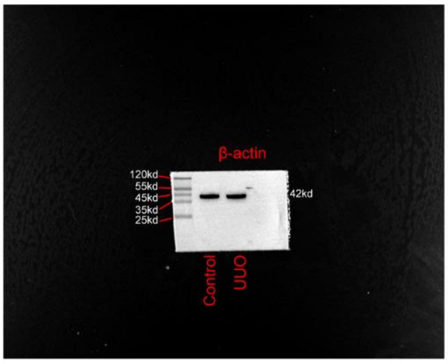

repeat_3

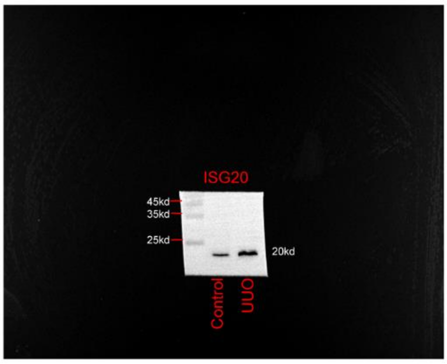

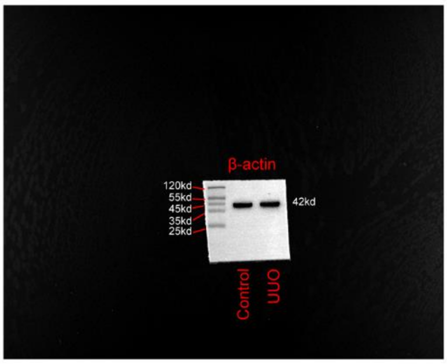

Original whole membrane of western blots in Fig4B

repeat_1: (Used in the manuscript)

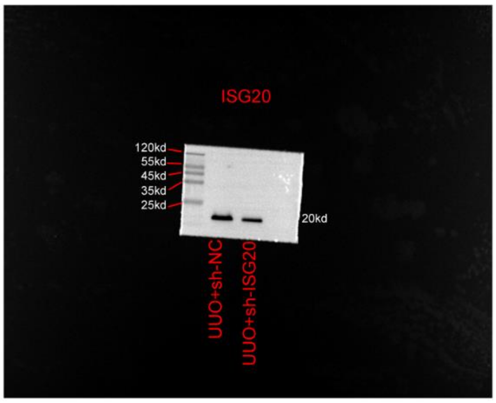

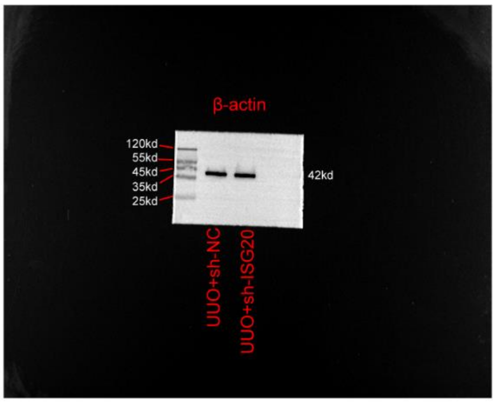

repeat_2

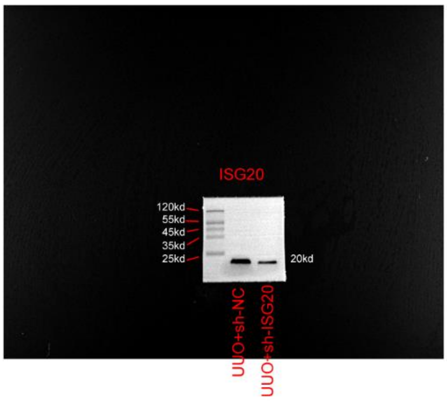

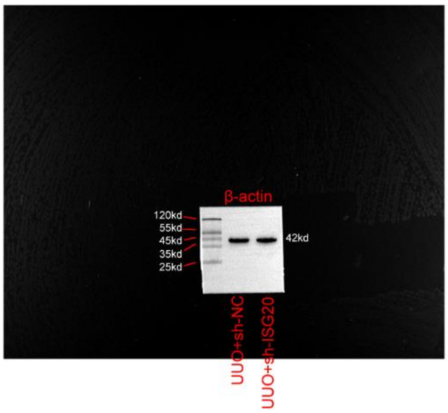

repeat_3

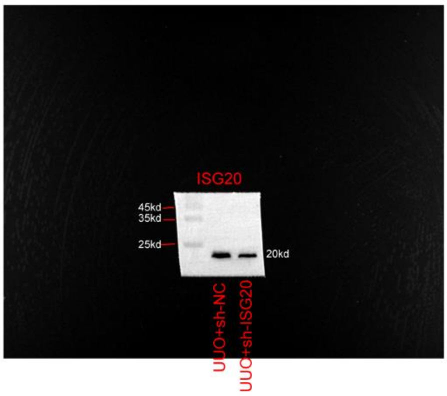

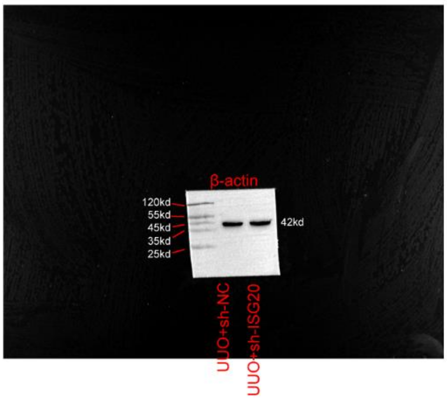

Original whole membrane of western blots in Fig4G

repeat_1: (Used in the manuscript)

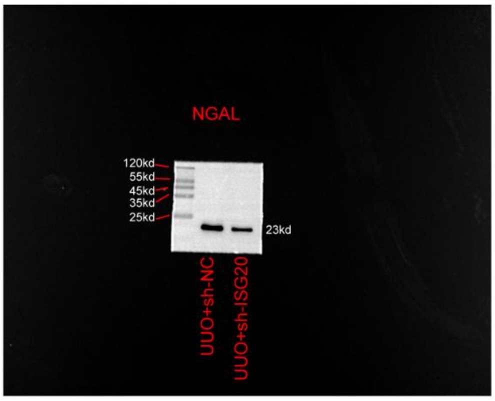

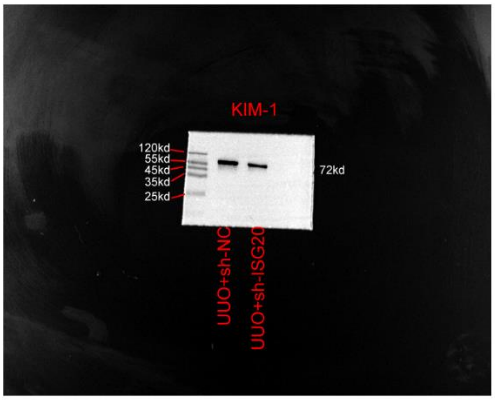

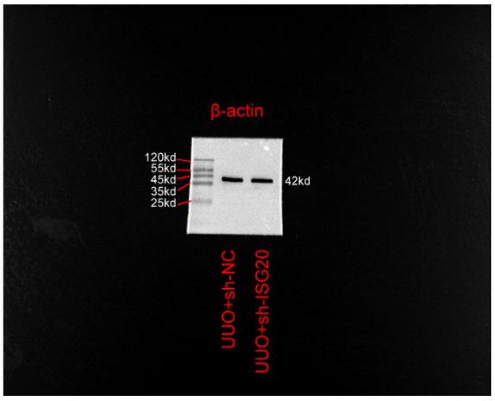

repeat_2

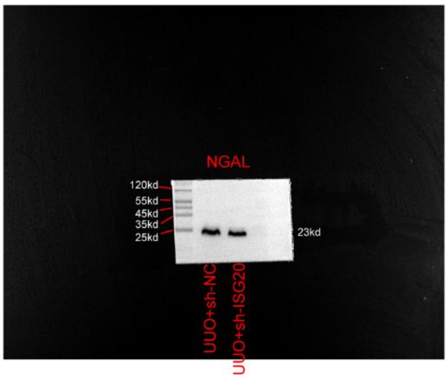

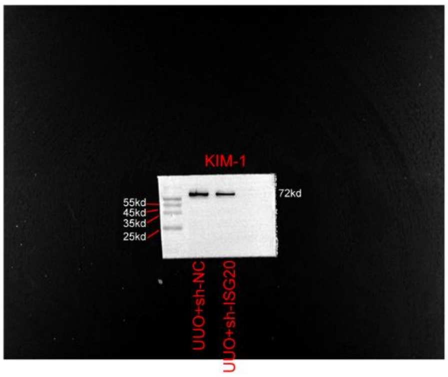

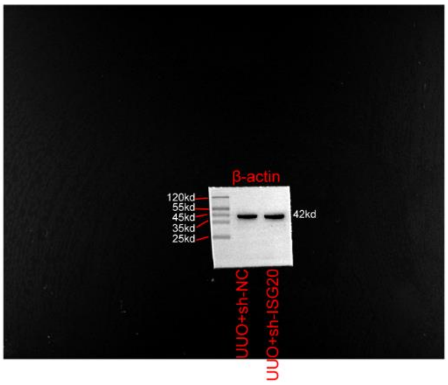

repeat_3

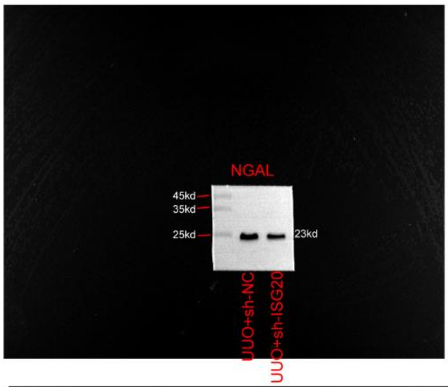
NGAL
45kd
35kd
25kd
23kd
UUO+sh-NC
UUO+sh-ISG20

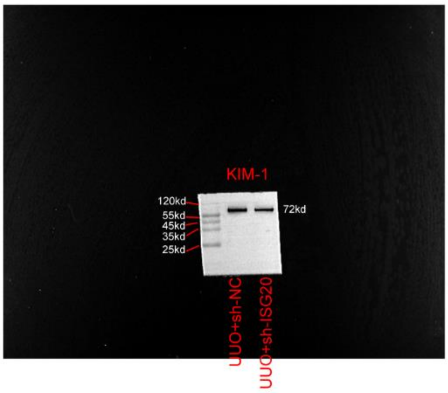
KIM-1
120kd
55kd
45kd
35kd
25kd
72kd
UUO+sh-NC
UUO+sh-ISG20

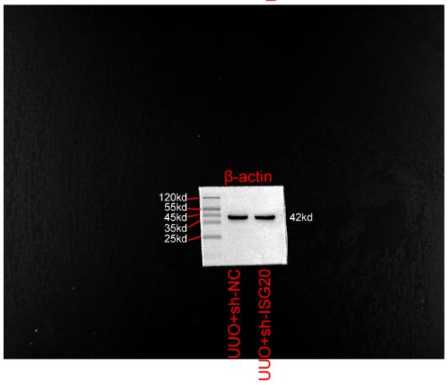
β-actin
120kd
55kd
45kd
35kd
25kd
42kd
UUO+sh-NC
UUO+sh-ISG20

Original whole membrane of western blots in Fig4H

repeat_1: (Used in the manuscript)

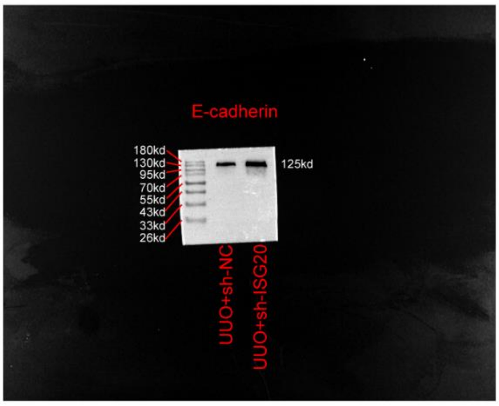

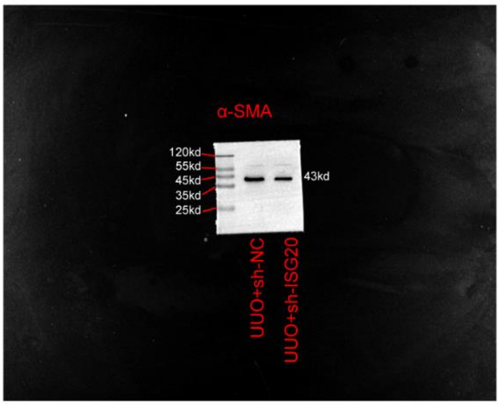

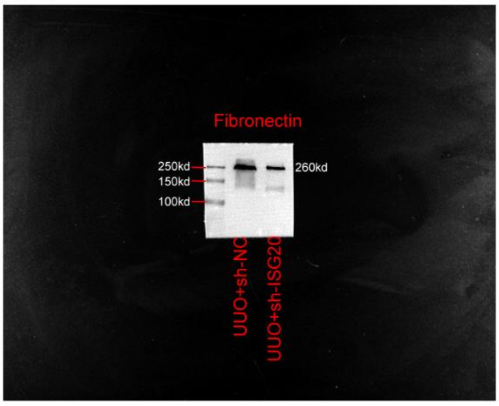

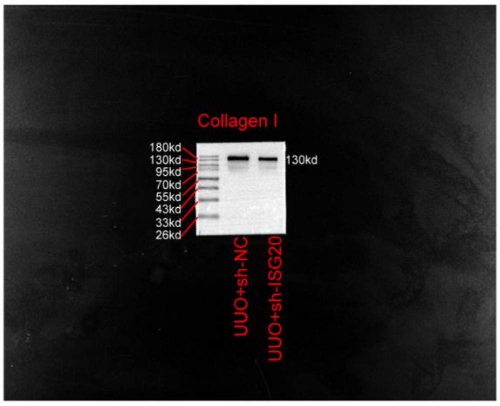

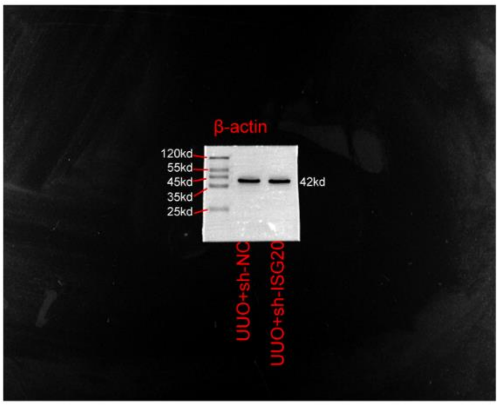

repeat_2

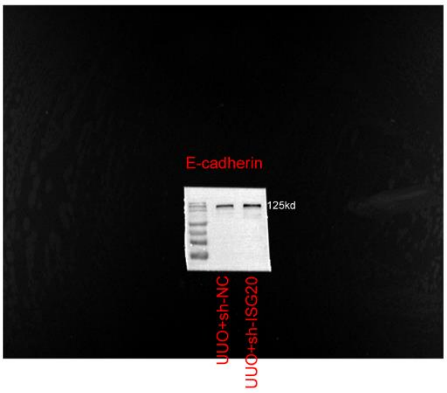
E-cadherin
125kd
UUO+sh-NC
UUO+sh-ISG20
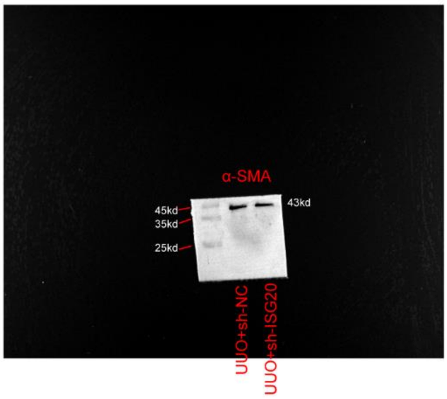
α-SMA
45kd
35kd
25kd
43kd
UUO+sh-NC
UUO+sh-ISG20
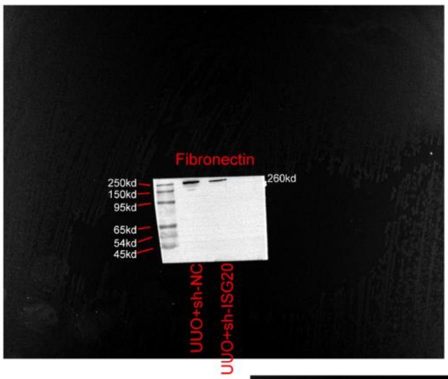
Fibronectin
250kd
150kd
95kd
65kd
54kd
45kd
260kd
UUO+sh-NC
UUO+sh-ISG20
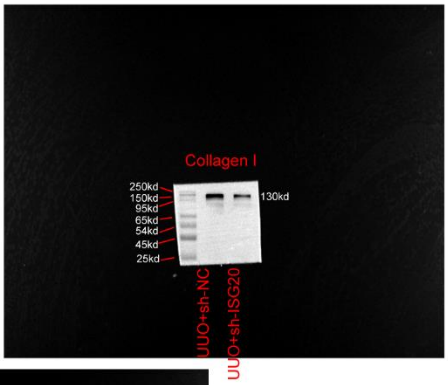
Collagen I
250kd
150kd
95kd
65kd
54kd
45kd
25kd
130kd
UUO+sh-NC
UUO+sh-ISG20
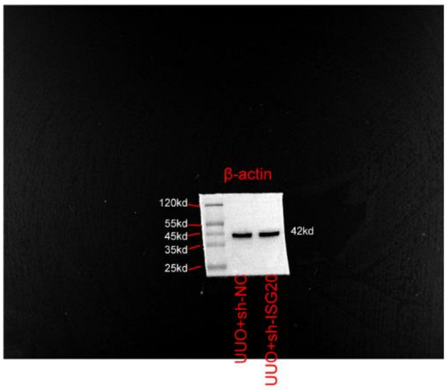
β-actin
120kd
55kd
45kd
35kd
25kd
42kd
UUO+sh-NC
UUO+sh-ISG20

repeat_3

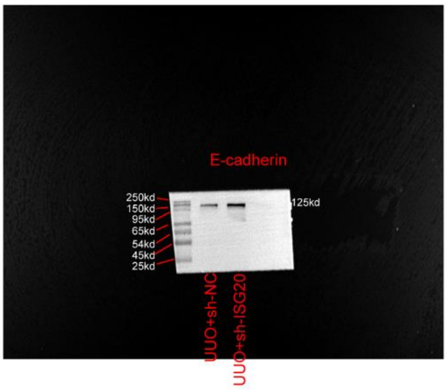
E-cadherin
250kd
150kd
95kd
65kd
54kd
45kd
25kd
125kd
UUO+sh-NC
UUO+sh-ISG20

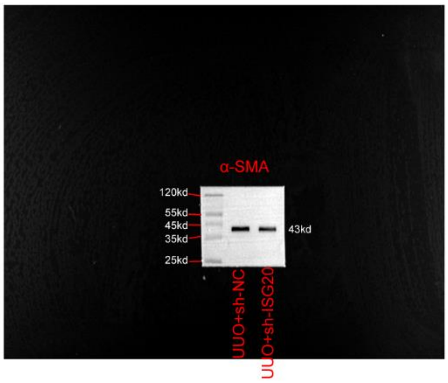
α-SMA
120kd
55kd
45kd
35kd
25kd
43kd
UUO+sh-NC
UUO+sh-ISG20

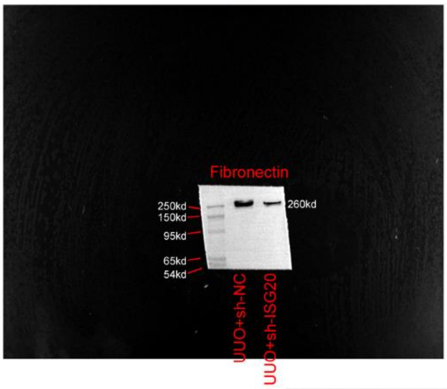
Fibronectin
250kd
150kd
95kd
65kd
54kd
260kd
UUO+sh-NC
UUO+sh-ISG20

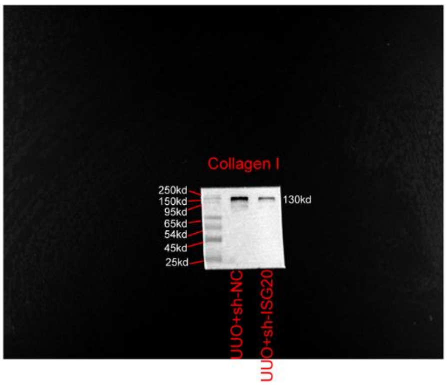
Collagen I
250kd
150kd
95kd
65kd
54kd
45kd
25kd
130kd
UUO+sh-NC
UUO+sh-ISG20

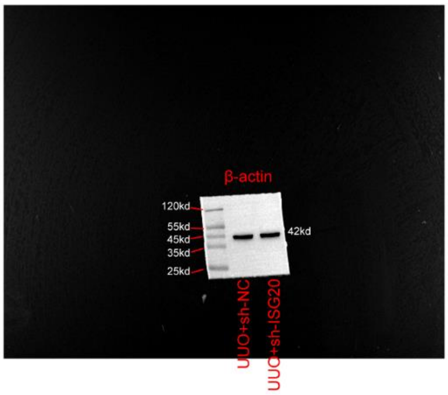
β-actin
120kd
55kd
45kd
35kd
25kd
42kd
UUO+sh-NC
UUO+sh-ISG20

Original whole membrane of western blots in Fig5A

repeat_1: (Used in the manuscript)

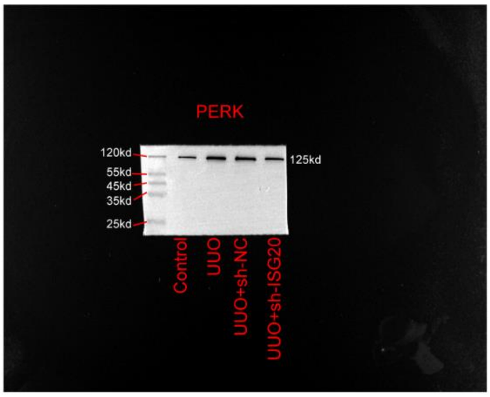

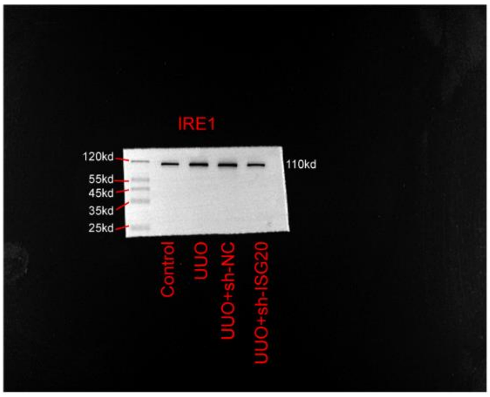

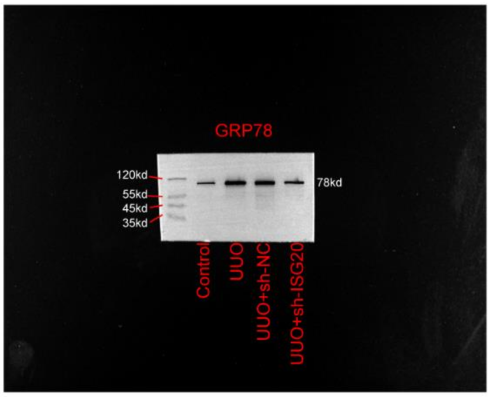

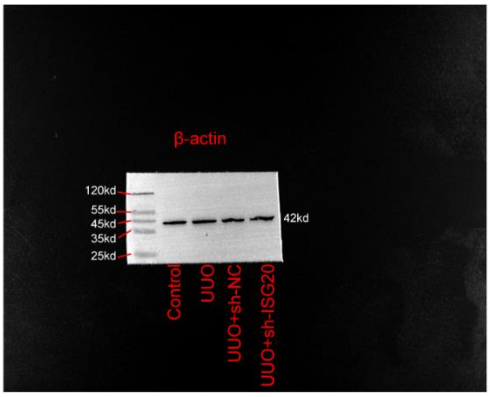

repeat_2

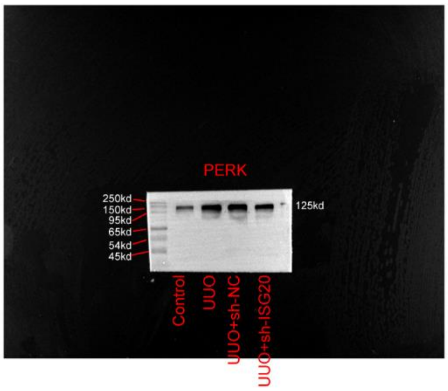
PERK
250kd
150kd
95kd
65kd
54kd
45kd
125kd
Control
UUO
UUO+sh-NC
UUO+sh-ISG20

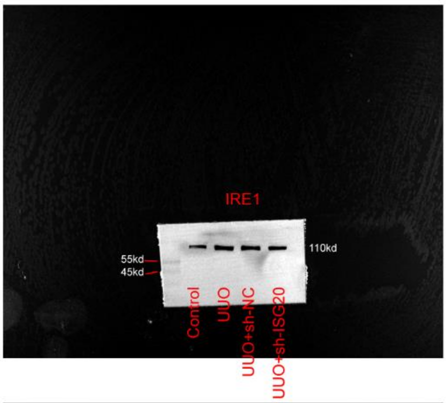
IRE1
55kd
45kd
110kd
Control
UUO
UUO+sh-NC
UUO+sh-ISG20

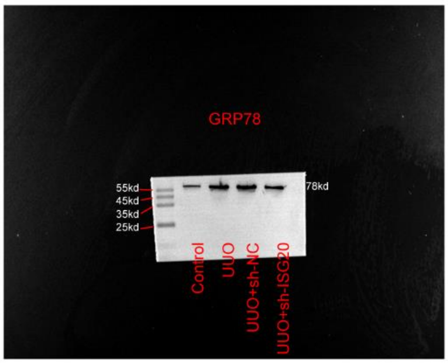
GRP78
55kd
45kd
35kd
25kd
78kd
Control
UUO
UUO+sh-NC
UUO+sh-ISG20

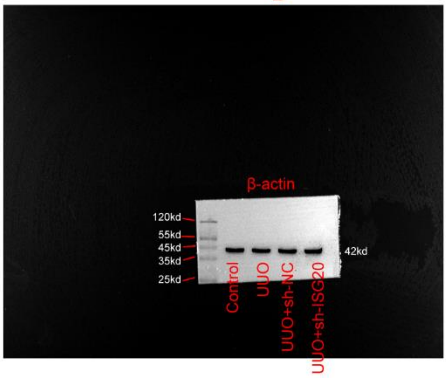
β-actin
120kd
55kd
45kd
35kd
25kd
42kd
Control
UUO
UUO+sh-NC
UUO+sh-ISG20

repeat_3

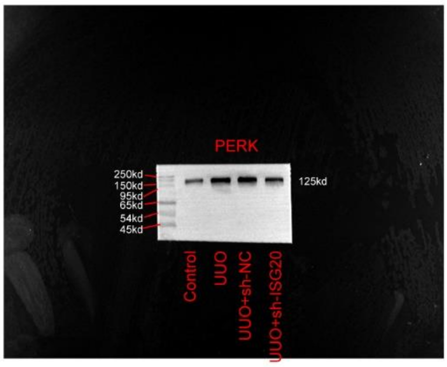
PERK
250kd
150kd
95kd
65kd
54kd
45kd
125kd
Control
UUO
UUO+sh-NC
UUO+sh-ISG20

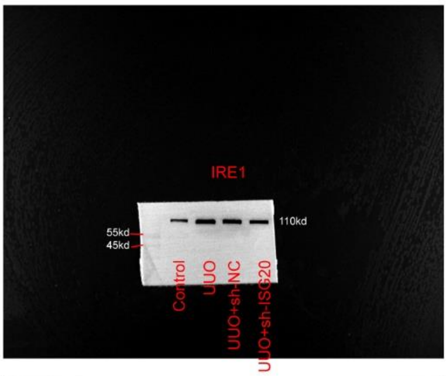
IRE1
55kd
45kd
110kd
Control
UUO
UUO+sh-NC
UUO+sh-ISG20

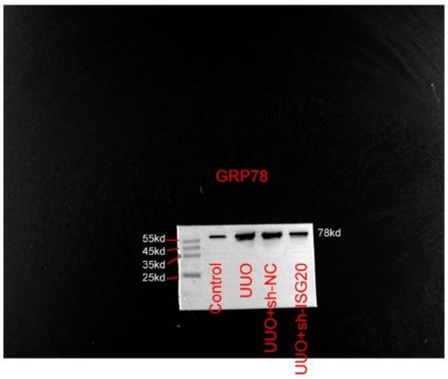
GRP78
55kd
45kd
35kd
25kd
78kd
Control
UUO
UUO+sh-NC
UUO+sh-ISG20

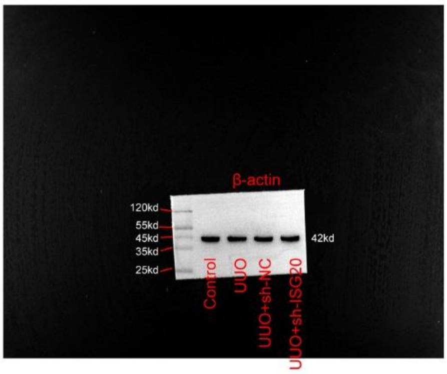
β-actin
120kd
55kd
45kd
35kd
25kd
42kd
Control
UUO
UUO+sh-NC
UUO+sh-ISG20

Original whole membrane of western blots in Fig5E

repeat_1: (Used in the manuscript)

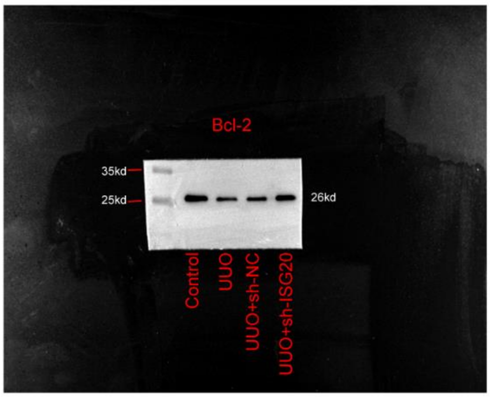

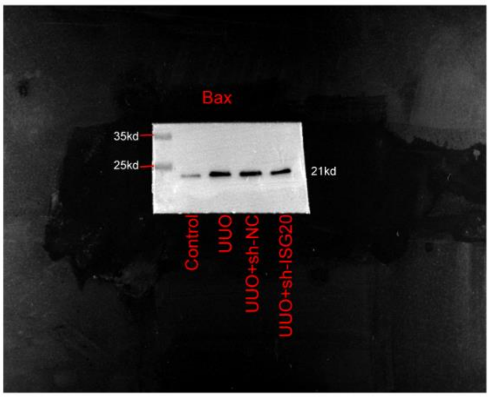

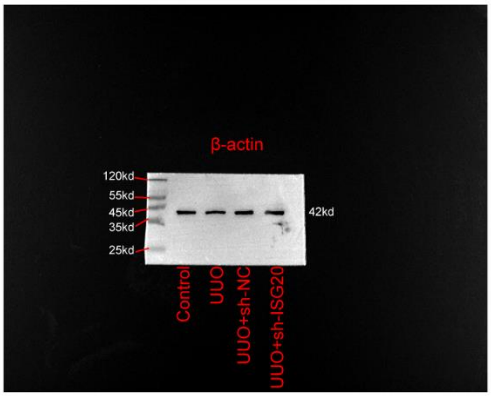

repeat_2

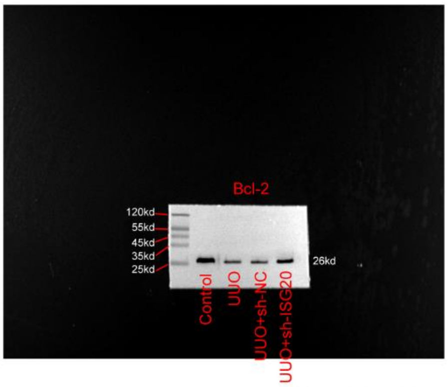

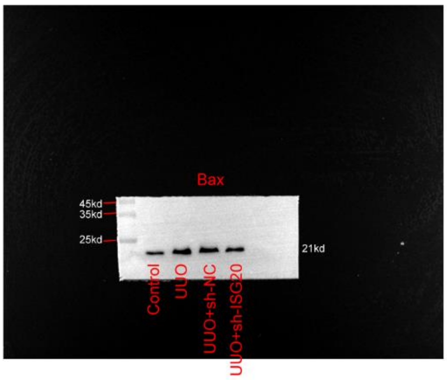

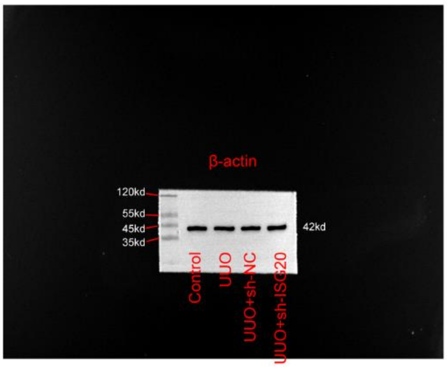

repeat_3

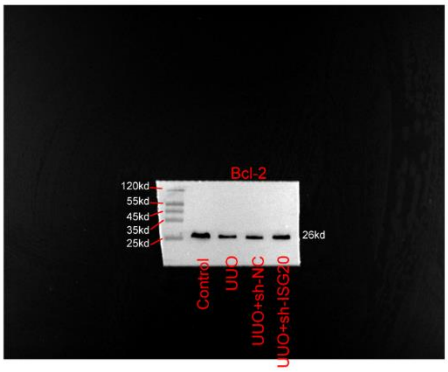
Bcl-2
120kd
55kd
45kd
35kd
25kd
26kd
Control
UUO
UUO+sh-NC
UUO+sh-ISG20

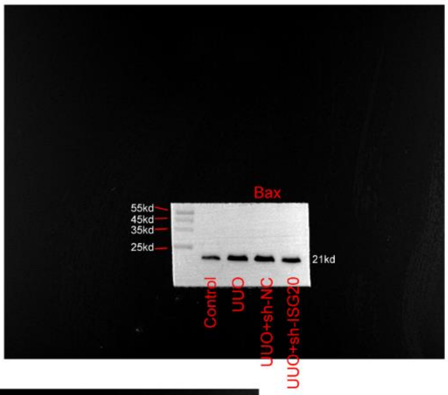
Bax
55kd
45kd
35kd
25kd
21kd
Control
UUO
UUO+sh-NC
UUO+sh-ISG20

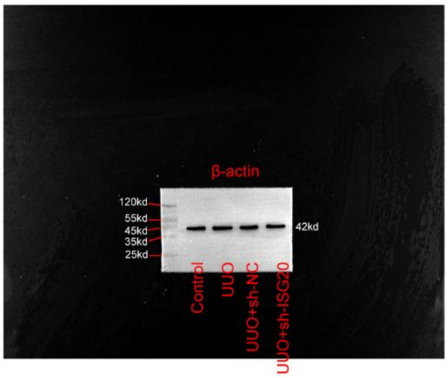
β-actin
120kd
55kd
45kd
35kd
25kd
42kd
Control
UUO
UUO+sh-NC
UUO+sh-ISG20
