# Supplementary material for: Targeting interferon-stimulated gene of 20 kDa protein (Isg20) inhibits ribosome biogenesis to ameliorate the progression of renal fibrosis
Source: PLoS One. 2025 Jul 7;20(7):e0322639. doi: 10.1371/journal.pone.0322639 (PMC12233288; doi:10.1371/journal.pone.0322639)
Supplement: S2 File — (PDF) [file pone.0322639.s002.pdf]

Fig2A

Descriptive Statistics

	N	Mean	Std. Deviation	Minimum	Maximum
SCr	12	51.8242	17.65897	22.32	82.18
BUN	12	9.0633	1.57607	6.96	12.10

One-Sample Kolmogorov-Smirnov Test

		SCr	BUN
N		12	12
Normal Parameters[a,b]	Mean	51.8242	9.0633
	Std. Deviation	17.65897	1.57607
Most Extreme Differences	Absolute	.151	.173
	Positive	.151	.173
	Negative	-.096	-.134
Kolmogorov-Smirnov Z		.525	.598
Asymp. Sig. (2-tailed)		.946	.867

a. Test distribution is Normal.

b. Calculated from data.

Group Statistics

	Group	N	Mean	Std. Deviation	Std. Error Mean
SCr	Control	6	38.0150	8.65904	3.53504
	UUO	6	65.6333	12.38564	5.05642
BUN	Control	6	7.9233	.69673	.28444
	UUO	6	10.2033	1.36393	.55682

Independent Samples Test

		Levene's Test for Equality of Variances		t-test for Equality of Means						
									95% Confidence Interval of the Difference	
		F	Sig.	t	df	Sig. (2-tailed)	Mean Difference	Std. Error Difference	Lower	Upper
SCr	Equal variances assumed	1.219	.296	-4.477	10	.001	-27.61833	6.16959	-41.36504	-13.87163
	Equal variances not assumed			-4.477	8.945	.002	-27.61833	6.16959	-41.58796	-13.64870
BUN	Equal variances assumed	6.721	.027	-3.646	10	.004	-2.28000	.62526	-3.67318	-.88682
	Equal variances not assumed			-3.646	7.443	.007	-2.28000	.62526	-3.74086	-.81914

Fig2E

Descriptive Statistics

	N	Mean	Std. Deviation	Minimum	Maximum
KIM1	6	.6350	.24139	.37	.88
NGAL	6	.6250	.39359	.23	1.00

One-Sample Kolmogorov-Smirnov Test

		KIM1	NGAL
N		6	6
Normal Parameters[a,b]	Mean	.6350	.6250
	Std. Deviation	.24139	.39359
Most Extreme Differences	Absolute	.290	.303
	Positive	.266	.288
	Negative	-.290	-.303
Kolmogorov-Smirnov Z		.711	.741
Asymp. Sig. (2-tailed)		.692	.642

a. Test distribution is Normal.

b. Calculated from data.

Group Statistics

	Group	N	Mean	Std. Deviation	Std. Error Mean
KIM1	Control	3	.4167	.04509	.02603
	UUO	3	.8533	.02517	.01453
NGAL	Control	3	.2667	.04041	.02333
	UUO	3	.9833	.02082	.01202

Independent Samples Test

		Levene's Test for Equality of Variances		t-test for Equality of Means						
									95% Confidence Interval of the Difference	
		F	Sig.	t	df	Sig. (2-tailed)	Mean Difference	Std. Error Difference	Lower	Upper
KIM1	Equal variances assumed	.720	.444	-14.646	4	.000	-.43667	.02981	-.51944	-.35389
	Equal variances not assumed			-14.646	3.136	.001	-.43667	.02981	-.52927	-.34407
NGAL	Equal variances assumed	1.180	.338	-27.305	4	.000	-.71667	.02625	-.78954	-.64379
	Equal variances not assumed			-27.305	2.991	.000	-.71667	.02625	-.80033	-.63300

Fig2F

Descriptive Statistics

	N	Mean	Std. Deviation	Minimum	Maximum
E.CADHERIN	6	.8983	.25270	.63	1.21
A.SMA	6	.7833	.29460	.47	1.09
FIBRONECTIN	6	.6317	.30275	.33	.95
COLLAGE.I	6	.7450	.25720	.48	1.02

One-Sample Kolmogorov-Smirnov Test

		E.CADHERIN	A.SMA	FIBRONECTIN	COLLAGE.I
N		6	6	6	6
Normal Parameters[a,b]	Mean	.8983	.7833	.6317	.7450
	Std. Deviation	.25270	.29460	.30275	.25720
Most Extreme Differences	Absolute	.260	.279	.297	.264
	Positive	.260	.276	.297	.264
	Negative	-.252	-.279	-.284	-.252
Kolmogorov-Smirnov Z		.636	.684	.728	.647
Asymp. Sig. (2-tailed)		.813	.738	.665	.797

a. Test distribution is Normal.

b. Calculated from data.

Group Statistics

	Group	N	Mean	Std. Deviation	Std. Error Mean
E.CADHERIN	Control	3	1.1233	.07572	.04372
	UUO	3	.6733	.04509	.02603
A.SMA	Control	3	.5167	.04509	.02603
	UUO	3	1.0500	.04000	.02309
FIBRONECTIN	Control	3	.3567	.02517	.01453
	UUO	3	.9067	.04041	.02333
COLLAGE.I	Control	3	.5133	.04163	.02404
	UUO	3	.9767	.05132	.02963

Independent Samples Test

		Levene's Test for Equality of Variances		t-test for Equality of Means						
									95% Confidence Interval of the Difference	
		F	Sig.	t	df	Sig. (2-tailed)	Mean Difference	Std. Error Difference	Lower	Upper
E.CADHERIN	Equal variances assumed	1.632	.271	8.844	4	.001	.45000	.05088	.30873	.59127
	Equal variances not assumed			8.844	3.260	.002	.45000	.05088	.29514	.60486
A.SMA	Equal variances assumed	.053	.829	-15.325	4	.000	-.53333	.03480	-.62996	-.43671
	Equal variances not assumed			-15.325	3.944	.000	-.53333	.03480	-.63050	-.43617
FIBRONECTIN	Equal variances assumed	.685	.454	-20.009	4	.000	-.55000	.02749	-.62632	-.47368
	Equal variances not assumed			-20.009	3.348	.000	-.55000	.02749	-.63255	-.46745
COLLAGE.I	Equal variances assumed	.172	.699	-12.144	4	.000	-.46333	.03815	-.56926	-.35741
	Equal variances not assumed			-12.144	3.837	.000	-.46333	.03815	-.57106	-.35561

Fig2G

Descriptive Statistics

	N	Mean	Std. Deviation	Minimum	Maximum
TNF.A	12	5.7917	4.29050	1.41	10.52
IL.1B	12	1.8775	.81007	1.00	2.84
IL.6	12	5.1092	2.51222	2.57	7.89

One-Sample Kolmogorov-Smirnov Test

		TNF.A	IL.1B	IL.6
N		12	12	12
Normal Parameters[a,b]	Mean	5.7917	1.8775	5.1092
	Std. Deviation	4.29050	.81007	2.51222
Most Extreme Differences	Absolute	.317	.288	.309
	Positive	.317	.288	.309
	Negative	-.270	-.271	-.305
Kolmogorov-Smirnov Z		1.097	.997	1.072
Asymp. Sig. (2-tailed)		.180	.273	.201

a. Test distribution is Normal.

b. Calculated from data.

Group Statistics

	Group	N	Mean	Std. Deviation	Std. Error Mean
TNF.A	Control	6	1.7033	.19336	.07894
	UUO	6	9.8800	.58852	.24026
IL.1B	Control	6	1.1083	.09368	.03825
	UUO	6	2.6467	.12258	.05004
IL.6	Control	6	2.7100	.13387	.05465
	UUO	6	7.5083	.22886	.09343

Independent Samples Test

		Levene's Test for Equality of Variances		t-test for Equality of Means						
									95% Confidence Interval of the Difference	
		F	Sig.	t	df	Sig. (2-tailed)	Mean Difference	Std. Error Difference	Lower	Upper
TNF.A	Equal variances assumed	4.326	.064	-32.332	10	.000	-8.17667	.25290	-8.74016	-7.61317
	Equal variances not assumed			-32.332	6.067	.000	-8.17667	.25290	-8.79384	-7.55950
IL.1B	Equal variances assumed	.207	.659	-24.423	10	.000	-1.53833	.06299	-1.67867	-1.39799
	Equal variances not assumed			-24.423	9.355	.000	-1.53833	.06299	-1.68000	-1.39667
IL.6	Equal variances assumed	.989	.343	-44.330	10	.000	-4.79833	.10824	-5.03951	-4.55716
	Equal variances not assumed			-44.330	8.063	.000	-4.79833	.10824	-5.04760	-4.54907

Fig2H

Descriptive Statistics

	N	Mean	Std. Deviation	Minimum	Maximum
rRNA.5S	12	2.2692	1.51335	.72	4.52
rRNA.5.8s	12	2.0225	1.19369	.41	3.82
rRNA.18s	12	1.8092	1.06911	.66	3.41
rRNA.28s	12	1.8675	.85816	1.00	3.69

One-Sample Kolmogorov-Smirnov Test

		rRNA.5S	rRNA.5.8s	rRNA.18s	rRNA.28s
N		12	12	12	12
Normal Parameters[a,b]	Mean	2.2692	2.0225	1.8092	1.8675
	Std. Deviation	1.51335	1.19369	1.06911	.85816
Most Extreme Differences	Absolute	.299	.204	.273	.271
	Positive	.299	.204	.273	.271
	Negative	-.162	-.132	-.177	-.156
Kolmogorov-Smirnov Z		1.036	.707	.944	.940
Asymp. Sig. (2-tailed)		.233	.699	.334	.341

a. Test distribution is Normal.

b. Calculated from data.

Group Statistics

	Group	N	Mean	Std. Deviation	Std. Error Mean
rRNA.5S	Control	6	.8900	.09209	.03759
	UUO	6	3.6483	.68189	.27838
rRNA.5.8s	Control	6	.9967	.35052	.14310
	UUO	6	3.0483	.69735	.28469
rRNA.18s	Control	6	.8683	.14525	.05930
	UUO	6	2.7500	.60752	.24802
rRNA.28s	Control	6	1.1533	.08238	.03363
	UUO	6	2.5817	.62394	.25472

Independent Samples Test

		Levene's Test for Equality of Variances		t-test for Equality of Means						
									95% Confidence Interval of the Difference	
		F	Sig.	t	df	Sig. (2-tailed)	Mean Difference	Std. Error Difference	Lower	Upper
rRNA.5S	Equal variances assumed	22.127	.001	-9.819	10	.000	-2.75833	.28091	-3.38424	-2.13243
	Equal variances not assumed			-9.819	5.182	.000	-2.75833	.28091	-3.47286	-2.04381
rRNA.5.8s	Equal variances assumed	3.868	.078	-6.439	10	.000	-2.05167	.31863	-2.76163	-1.34171
	Equal variances not assumed			-6.439	7.375	.000	-2.05167	.31863	-2.79742	-1.30592
rRNA.18s	Equal variances assumed	10.990	.008	-7.379	10	.000	-1.88167	.25501	-2.44986	-1.31347
	Equal variances not assumed			-7.379	5.570	.000	-1.88167	.25501	-2.51752	-1.24581
rRNA.28s	Equal variances assumed	4.333	.064	-5.559	10	.000	-1.42833	.25693	-2.00081	-.85585
	Equal variances not assumed			-5.559	5.174	.002	-1.42833	.25693	-2.08216	-.77450

Fig3D

Descriptive Statistics

	N	Mean	Std. Deviation	Minimum	Maximum
Rps19	12	.9561	.19746	.72	1.32
Isg20	12	1.6278	.69687	.80	2.63
Rps9	12	1.2087	.30542	.77	1.88

One-Sample Kolmogorov-Smirnov Test

		Rps19	Isg20	Rps9
N		12	12	12
Normal Parameters[a,b]	Mean	.9561	1.6278	1.2087
	Std. Deviation	.19746	.69687	.30542
Most Extreme Differences	Absolute	.177	.230	.152
	Positive	.177	.230	.152
	Negative	-.119	-.171	-.080
Kolmogorov-Smirnov Z		.613	.796	.526
Asymp. Sig. (2-tailed)		.847	.551	.945

a. Test distribution is Normal.

b. Calculated from data.

Group Statistics

	Group	N	Mean	Std. Deviation	Std. Error Mean
Rps19	Control	6	.9365	.14281	.05830
	UUO	6	.9757	.25389	.10365
Isg20	Control	6	.9957	.20707	.08454
	UUO	6	2.2600	.25784	.10526
Rps9	Control	6	.9944	.17284	.07056
	UUO	6	1.4230	.25520	.10418

Independent Samples Test

		Levene's Test for Equality of Variances		t-test for Equality of Means						
									95% Confidence Interval of the Difference	
		F	Sig.	t	df	Sig. (2-tailed)	Mean Difference	Std. Error Difference	Lower	Upper
Rps19	Equal variances assumed	3.610	.087	-.329	10	.749	-.03918	.11892	-.30416	.22580
	Equal variances not assumed			-.329	7.876	.750	-.03918	.11892	-.31417	.23581
Isg20	Equal variances assumed	.578	.465	-9.364	10	.000	-1.26427	.13501	-1.56508	-.96345
	Equal variances not assumed			-9.364	9.555	.000	-1.26427	.13501	-1.56699	-.96154
Rps9	Equal variances assumed	.545	.477	-3.406	10	.007	-.42860	.12583	-.70896	-.14823
	Equal variances not assumed			-3.406	8.790	.008	-.42860	.12583	-.71429	-.14291

Fig3E

Descriptive Statistics

	N	Mean	Std. Deviation	Minimum	Maximum
ISG20	6	.8218	.28465	.54	1.14

One-Sample Kolmogorov-Smirnov Test

		ISG20
N		6
Normal Parameters[a,b]	Mean	.8218
	Std. Deviation	.28465
Most Extreme Differences	Absolute	.285
	Positive	.285
	Negative	-.266
Kolmogorov-Smirnov Z		.699
Asymp. Sig. (2-tailed)		.712

a. Test distribution is Normal.

b. Calculated from data.

Group Statistics

	Group	N	Mean	Std. Deviation	Std. Error Mean
ISG20	Control	3	.5645	.02815	.01625
	UUO	3	1.0792	.05520	.03187

Independent Samples Test

		Levene's Test for Equality of Variances		t-test for Equality of Means					95% Confidence Interval of the Difference	
		F	Sig.	t	df	Sig. (2-tailed)	Mean Difference	Std. Error Difference	Lower	Upper
ISG20	Equal variances assumed	1.139	.346	-14.388	4	.000	-.51474	.03578	-.61407	-.41541
	Equal variances not assumed			-14.388	2.975	.001	-.51474	.03578	-.62915	-.40034

Fig3F

Descriptive Statistics

	N	Mean	Std. Deviation	Minimum	Maximum
AOD	6	.1867	.05922	.11	.26

One-Sample Kolmogorov-Smirnov Test

		AOD
N		6
Normal Parameters[a,b]	Mean	.1867
	Std. Deviation	.05922
Most Extreme Differences	Absolute	.174
	Positive	.174
	Negative	-.153
Kolmogorov-Smirnov Z		.426
Asymp. Sig. (2-tailed)		.994

a. Test distribution is Normal.

b. Calculated from data.

Group Statistics

	Group	N	Mean	Std. Deviation	Std. Error Mean
AOD	Control	3	.1367	.02517	.01453
	UUO	3	.2367	.02517	.01453

Independent Samples Test

		Levene's Test for Equality of Variances		t-test for Equality of Means						
									95% Confidence Interval of the Difference	
		F	Sig.	t	df	Sig. (2-tailed)	Mean Difference	Std. Error Difference	Lower	Upper
AOD	Equal variances assumed	.000	1.000	-4.867	4	.008	-.10000	.02055	-.15705	-.04295
	Equal variances not assumed			-4.867	4.000	.008	-.10000	.02055	-.15705	-.04295

Fig4A

Descriptive Statistics

	N	Mean	Std. Deviation	Minimum	Maximum
ISG20	12	.7227	.35789	.20	1.18

One-Sample Kolmogorov-Smirnov Test

		ISG20
N		12
Normal Parameters[a,b]	Mean	.7227
	Std. Deviation	.35789
Most Extreme Differences	Absolute	.161
	Positive	.161
	Negative	-.155
Kolmogorov-Smirnov Z		.559
Asymp. Sig. (2-tailed)		.913

a. Test distribution is Normal.

b. Calculated from data.

Group Statistics

	Group	N	Mean	Std. Deviation	Std. Error Mean
ISG20	UUO+sh-NC	6	1.0422	.13646	.05571
	UUO+sh-Isg20	6	.4032	.13490	.05507

Independent Samples Test

		Levene's Test for Equality of Variances		t-test for Equality of Means						
									95% Confidence Interval of the Difference	
		F	Sig.	t	df	Sig. (2-tailed)	Mean Difference	Std. Error Difference	Lower	Upper
ISG20	Equal variances assumed	.322	.583	8.157	10	.000	.63896	.07833	.46442	.81350
	Equal variances not assumed			8.157	9.999	.000	.63896	.07833	.46442	.81350

Fig4B

Descriptive Statistics

	N	Mean	Std. Deviation	Minimum	Maximum
ISG20	6	.8394	.18411	.64	1.04

One-Sample Kolmogorov-Smirnov Test

		ISG20
N		6
Normal Parameters[a,b]	Mean	.8394
	Std. Deviation	.18411
Most Extreme Differences	Absolute	.271
	Positive	.271
	Negative	-.266
Kolmogorov-Smirnov Z		.664
Asymp. Sig. (2-tailed)		.771

a. Test distribution is Normal.

b. Calculated from data.

Group Statistics

	Group	N	Mean	Std. Deviation	Std. Error Mean
ISG20	UUO+sh-NC	3	1.0052	.03595	.02076
	UUO+sh-Isg20	3	.6737	.03161	.01825

Independent Samples Test

		Levene's Test for Equality of Variances		t-test for Equality of Means						
									95% Confidence Interval of the Difference	
		F	Sig.	t	df	Sig. (2-tailed)	Mean Difference	Std. Error Difference	Lower	Upper
ISG20	Equal variances assumed	.068	.807	11.996	4	.000	.33157	.02764	.25482	.40831
	Equal variances not assumed			11.996	3.936	.000	.33157	.02764	.25433	.40881

Fig4C

Descriptive Statistics

	N	Mean	Std. Deviation	Minimum	Maximum
SCr	12	60.5911	17.53106	29.99	81.91
BUN	12	8.0761	1.29098	6.19	9.99

One-Sample Kolmogorov-Smirnov Test

		SCr	BUN
N		12	12
Normal Parameters[a,b]	Mean	60.5911	8.0761
	Std. Deviation	17.53106	1.29098
Most Extreme Differences	Absolute	.153	.131
	Positive	.123	.126
	Negative	-.153	-.131
Kolmogorov-Smirnov Z		.532	.455
Asymp. Sig. (2-tailed)		.940	.986

a. Test distribution is Normal.

b. Calculated from data.

Group Statistics

	Group	N	Mean	Std. Deviation	Std. Error Mean
SCr	UUO+sh-NC	6	74.7652	8.92715	3.64449
	UUO+sh-lsg20	6	46.4171	10.69032	4.36430
BUN	UUO+sh-NC	6	8.9225	.94576	.38611
	UUO+sh-lsg20	6	7.2296	1.02599	.41886

Independent Samples Test

		Levene's Test for Equality of Variances		t-test for Equality of Means						
									95% Confidence Interval of the Difference	
		F	Sig.	t	df	Sig. (2-tailed)	Mean Difference	Std. Error Difference	Lower	Upper
SCr	Equal variances assumed	.075	.790	4.986	10	.001	28.34804	5.68590	15.67906	41.01702
	Equal variances not assumed			4.986	9.692	.001	28.34804	5.68590	15.62425	41.07184
BUN	Equal variances assumed	.613	.452	2.972	10	.014	1.69288	.56967	.42358	2.96217
	Equal variances not assumed			2.972	9.934	.014	1.69288	.56967	.42245	2.96331

Fig4G

Descriptive Statistics

	N	Mean	Std. Deviation	Minimum	Maximum
KIM.1	6	.7961	.12837	.63	.95
NGAL	6	.8750	.20335	.65	1.10

One-Sample Kolmogorov-Smirnov Test

		KIM.1	NGAL
N		6	6
Normal Parameters[a,b]	Mean	.7961	.8750
	Std. Deviation	.12837	.20335
Most Extreme Differences	Absolute	.252	.260
	Positive	.194	.257
	Negative	-.252	-.260
Kolmogorov-Smirnov Z		.616	.637
Asymp. Sig. (2-tailed)		.842	.811

a. Test distribution is Normal.

b. Calculated from data.

Group Statistics

	Group	N	Mean	Std. Deviation	Std. Error Mean
KIM.1	UUO+sh-NC	3	.9075	.03466	.02001
	UUO+sh-lsg20	3	.6847	.05267	.03041
NGAL	UUO+sh-NC	3	1.0575	.04126	.02382
	UUO+sh-lsg20	3	.6925	.04162	.02403

Independent Samples Test

		Levene's Test for Equality of Variances		t-test for Equality of Means						
									95% Confidence Interval of the Difference	
		F	Sig.	t	df	Sig. (2-tailed)	Mean Difference	Std. Error Difference	Lower	Upper
KIM.1	Equal variances assumed	.569	.493	6.120	4	.004	.22278	.03640	.12171	.32386
	Equal variances not assumed			6.120	3.458	.006	.22278	.03640	.11515	.33042
NGAL	Equal variances assumed	.001	.978	10.788	4	.000	.36505	.03384	.27110	.45900
	Equal variances not assumed			10.788	4.000	.000	.36505	.03384	.27110	.45900

Fig4H

Descriptive Statistics

	N	Mean	Std. Deviation	Minimum	Maximum
E.CADHERIN	6	.7791	.24741	.51	1.07
a.SMA	6	.7461	.14892	.59	.92
FIBRONECTIN	6	.7024	.24225	.45	.96
COLLAGE.I	6	.8221	.12989	.67	.97

One-Sample Kolmogorov-Smirnov Test

		E.CADHERIN	a.SMA	FIBRONECTIN	COLLAGE.I
N		6	6	6	6
Normal Parameters[a,b]	Mean	.7791	.7461	.7024	.8221
	Std. Deviation	.24741	.14892	.24225	.12989
Most Extreme Differences	Absolute	.267	.212	.284	.184
	Positive	.267	.212	.284	.172
	Negative	-.245	-.203	-.267	-.184
Kolmogorov-Smirnov Z		.654	.519	.697	.451
Asymp. Sig. (2-tailed)		.785	.951	.717	.987

a. Test distribution is Normal.

b. Calculated from data.

Group Statistics

	Group	N	Mean	Std. Deviation	Std. Error Mean
E.CADHERIN	UUO+sh-NC	3	.5576	.04396	.02538
	UUO+sh-Isg20	3	1.0007	.06180	.03568
a.SMA	UUO+sh-NC	3	.8769	.04974	.02872
	UUO+sh-Isg20	3	.6154	.04129	.02384
FIBRONECTIN	UUO+sh-NC	3	.9216	.04061	.02345
	UUO+sh-Isg20	3	.4832	.02937	.01696
COLLAGE.I	UUO+sh-NC	3	.9343	.04516	.02607
	UUO+sh-Isg20	3	.7099	.04897	.02827

Independent Samples Test

		Levene's Test for Equality of Variances		t-test for Equality of Means						
									95% Confidence Interval of the Difference	
		F	Sig.	t	df	Sig. (2-tailed)	Mean Difference	Std. Error Difference	Lower	Upper
E.CADHERIN	Equal variances assumed	.285	.622	-10.121	4	.001	-.44314	.04379	-.56471	-.32157
	Equal variances not assumed			-10.121	3.611	.001	-.44314	.04379	-.57004	-.31623
a.SMA	Equal variances assumed	.021	.891	7.005	4	.002	.26144	.03732	.15782	.36507
	Equal variances not assumed			7.005	3.869	.002	.26144	.03732	.15642	.36647
FIBRONECTIN	Equal variances assumed	.285	.622	15.153	4	.000	.43848	.02894	.35814	.51882
	Equal variances not assumed			15.153	3.642	.000	.43848	.02894	.35493	.52203
COLLAGE.I	Equal variances assumed	.047	.840	5.833	4	.004	.22432	.03846	.11754	.33110
	Equal variances not assumed			5.833	3.974	.004	.22432	.03846	.11726	.33138

Fig4I

Descriptive Statistics

	N	Mean	Std. Deviation	Minimum	Maximum
TNF.a	12	7.2953	1.97281	5.21	10.54
IL.1b	12	2.2565	.30800	1.87	2.70
IL.6	12	6.0355	1.45095	4.08	7.64

One-Sample Kolmogorov-Smirnov Test

		TNF.a	IL.1b	IL.6
N		12	12	12
Normal Parameters[a,b]	Mean	7.2953	2.2565	6.0355
	Std. Deviation	1.97281	.30800	1.45095
Most Extreme Differences	Absolute	.268	.188	.250
	Positive	.268	.188	.191
	Negative	-.191	-.164	-.250
Kolmogorov-Smirnov Z		.928	.652	.867
Asymp. Sig. (2-tailed)		.356	.789	.440

a. Test distribution is Normal.

b. Calculated from data.

Group Statistics

	Group	N	Mean	Std. Deviation	Std. Error Mean
TNF.a	UUO+sh-NC	6	9.0779	.93991	.38372
	UUO+sh-lsg20	6	5.5127	.22998	.09389
IL.1b	UUO+sh-NC	6	2.5339	.11594	.04733
	UUO+sh-lsg20	6	1.9790	.10234	.04178
IL.6	UUO+sh-NC	6	7.3722	.24787	.10119
	UUO+sh-lsg20	6	4.6989	.53116	.21684

Independent Samples Test

		Levene's Test for Equality of Variances		t-test for Equality of Means						
									95% Confidence Interval of the Difference	
		F	Sig.	t	df	Sig. (2-tailed)	Mean Difference	Std. Error Difference	Lower	Upper
TNF.a	Equal variances assumed	11.567	.007	9.025	10	.000	3.56513	.39504	2.68493	4.44532
	Equal variances not assumed			9.025	5.597	.000	3.56513	.39504	2.58136	4.54889
IL.1b	Equal variances assumed	.017	.898	8.790	10	.000	.55496	.06314	.41428	.69563
	Equal variances not assumed			8.790	9.848	.000	.55496	.06314	.41399	.69593
IL.6	Equal variances assumed	9.501	.012	11.172	10	.000	2.67333	.23929	2.14016	3.20651
	Equal variances not assumed			11.172	7.079	.000	2.67333	.23929	2.10878	3.23789

Fig4J

Descriptive Statistics

	N	Mean	Std. Deviation	Minimum	Maximum
rRNA.5s	12	.7238	.33975	.29	1.28
rRNA.5.8s	12	.7022	.41047	.19	1.33
rRNA.18s	12	.8089	.34466	.31	1.35
rRNA.28s	12	.6673	.24237	.30	1.03

One-Sample Kolmogorov-Smirnov Test

		rRNA.5s	rRNA.5.8s	rRNA.18s	rRNA.28s
N		12	12	12	12
Normal Parameters[a,b]	Mean	.7238	.7022	.8089	.6673
	Std. Deviation	.33975	.41047	.34466	.24237
Most Extreme Differences	Absolute	.190	.259	.134	.191
	Positive	.190	.259	.134	.191
	Negative	-.146	-.185	-.128	-.149
Kolmogorov-Smirnov Z		.658	.896	.465	.660
Asymp. Sig. (2-tailed)		.779	.398	.982	.776

Group Statistics

	Group	N	Mean	Std. Deviation	Std. Error Mean
rRNA.5s	UUO+sh-NC	6	1.0219	.16905	.06902
	UUO+sh-Isg20	6	.4256	.10967	.04477
rRNA.5.8s	UUO+sh-NC	6	1.0794	.15210	.06209
	UUO+sh-Isg20	6	.3250	.07788	.03179
rRNA.18s	UUO+sh-NC	6	1.1002	.19970	.08153
	UUO+sh-Isg20	6	.5177	.13356	.05453
rRNA.28s	UUO+sh-NC	6	.8702	.15014	.06129
	UUO+sh-Isg20	6	.4645	.08923	.03643

Independent Samples Test

		Levene's Test for Equality of Variances		t-test for Equality of Means						
									95% Confidence Interval of the Difference	
		F	Sig.	t	df	Sig. (2-tailed)	Mean Difference	Std. Error Difference	Lower	Upper
rRNA.5s	Equal variances assumed	.384	.549	7.248	10	.000	.59629	.08227	.41298	.77959
	Equal variances not assumed			7.248	8.575	.000	.59629	.08227	.40877	.78380
rRNA.5.8s	Equal variances assumed	1.067	.326	10.814	10	.000	.75440	.06976	.59897	.90984
	Equal variances not assumed			10.814	7.453	.000	.75440	.06976	.59146	.91735
rRNA.18s	Equal variances assumed	.596	.458	5.940	10	.000	.58255	.09808	.36402	.80109
	Equal variances not assumed			5.940	8.727	.000	.58255	.09808	.35962	.80549
rRNA.28s	Equal variances assumed	.868	.373	5.689	10	.000	.40566	.07130	.24679	.56453
	Equal variances not assumed			5.689	8.140	.000	.40566	.07130	.24172	.56959

Fig5A

Descriptive Statistics

	N	Mean	Std. Deviation	Minimum	Maximum
PERK	12	.7078	.19350	.42	.93
IRE1	12	.8422	.13952	.65	1.03
GRP78	12	.7669	.25184	.41	1.06

One-Sample Kolmogorov-Smirnov Test

		PERK	IRE1	GRP78
N		12	12	12
Normal Parameters[a,b]	Mean	.7078	.8422	.7669
	Std. Deviation	.19350	.13952	.25184
Most Extreme Differences	Absolute	.248	.157	.262
	Positive	.134	.146	.155
	Negative	-.248	-.157	-.262
Kolmogorov-Smirnov Z		.859	.542	.907
Asymp. Sig. (2-tailed)		.452	.930	.383

a. Test distribution is Normal.

b. Calculated from data.

Test of Homogeneity of Variances

	Levene Statistic	df1	df2	Sig.
PERK	.437	3	8	.732
IRE1	.184	3	8	.905
GRP78	.787	3	8	.534

ANOVA

		Sum of Squares	df	Mean Square	F	Sig.
PERK	Between Groups	.402	3	.134	109.584	.000
	Within Groups	.010	8	.001		
	Total	.412	11			
IRE1	Between Groups	.191	3	.064	22.381	.000
	Within Groups	.023	8	.003		
	Total	.214	11			
GRP78	Between Groups	.682	3	.227	116.248	.000
	Within Groups	.016	8	.002		
	Total	.698	11			

Multiple Comparisons

Dependent Variable		(I) Group	(J) Group	Mean Difference (I-J)	Std. Error	Sig.	95% Confidence Interval	
							Lower Bound	Upper Bound
PERK	LSD	Control	UUO	-.41102*	.02855	.000	-.4769	-.3452
			UUO+sh-NC	-.45388*	.02855	.000	-.5197	-.3880
			UUO+sh-Isg20	-.18228*	.02855	.000	-.2481	-.1164
		UUO	Control	.41102*	.02855	.000	.3452	.4769
			UUO+sh-NC	-.04285	.02855	.172	-.1087	.0230
			UUO+sh-Isg20	.22874*	.02855	.000	.1629	.2946
		UUO+sh-NC	Control	.45388*	.02855	.000	.3880	.5197
			UUO	.04285	.02855	.172	-.0230	.1087
			UUO+sh-Isg20	.27160*	.02855	.000	.2058	.3374
		UUO+sh-Isg20	Control	.18228*	.02855	.000	.1164	.2481
			UUO	-.22874*	.02855	.000	-.2946	-.1629
			UUO+sh-NC	-.27160*	.02855	.000	-.3374	-.2058
	Dunnett T3	Control	UUO	-.41102*	.02159	.000	-.5067	-.3153
			UUO+sh-NC	-.45388*	.02544	.000	-.5665	-.3413
			UUO+sh-Isg20	-.18228*	.03258	.035	-.3440	-.0205
		UUO	Control	.41102*	.02159	.000	.3153	.5067
			UUO+sh-NC	-.04285	.02386	.492	-.1528	.0671
			UUO+sh-Isg20	.22874*	.03136	.021	.0616	.3959
		UUO+sh-NC	Control	.45388*	.02544	.000	.3413	.5665
			UUO	.04285	.02386	.492	-.0671	.1528
			UUO+sh-Isg20	.27160*	.03413	.009	.1121	.4311
		UUO+sh-Isg20	Control	.18228*	.03258	.035	.0205	.3440
			UUO	-.22874*	.03136	.021	-.3959	-.0616
			UUO+sh-NC	-.27160*	.03413	.009	-.4311	-.1121
IRE1	LSD	Control	UUO	-.27159*	.04359	.000	-.3721	-.1711
			UUO+sh-NC	-.28872*	.04359	.000	-.3892	-.1882
			UUO+sh-Isg20	-.06397	.04359	.180	-.1645	.0365
		UUO	Control	.27159*	.04359	.000	.1711	.3721
			UUO+sh-NC	-.01713	.04359	.705	-.1176	.0834
			UUO+sh-Isg20	.20761*	.04359	.001	.1071	.3081
		UUO+sh-NC	Control	.28872*	.04359	.000	.1882	.3892
			UUO	.01713	.04359	.705	-.0834	.1176
			UUO+sh-Isg20	.22474*	.04359	.001	.1242	.3253
		UUO+sh-Isg20	Control	.06397	.04359	.180	-.0365	.1645
			UUO	-.20761*	.04359	.001	-.3081	-.1071
			UUO+sh-NC	-.22474*	.04359	.001	-.3253	-.1242
	Dunnett T3	Control	UUO	-.27159*	.04298	.017	-.4662	-.0770
			UUO+sh-NC	-.28872*	.04349	.014	-.4866	-.0908
			UUO+sh-Isg20	-.06397	.03779	.531	-.2291	.1012
		UUO	Control	.27159*	.04298	.017	.0770	.4662
			UUO+sh-NC	-.01713	.04870	.999	-.2298	.1955
			UUO+sh-Isg20	.20761*	.04368	.041	.0119	.4034
		UUO+sh-NC	Control	.28872*	.04349	.014	.0908	.4866
			UUO	.01713	.04870	.999	-.1955	.2298
			UUO+sh-Isg20	.22474*	.04418	.034	.0259	.4236
		UUO+sh-Isg20	Control	.06397	.03779	.531	-.1012	.2291
			UUO	-.20761*	.04368	.041	-.4034	-.0119
			UUO+sh-NC	-.22474*	.04418	.034	-.4236	-.0259

GRP78	LSD	Control	UUO	-.55149*	.03611	.000	-.6348	-.4682
			UUO+sh-NC	-.55752*	.03611	.000	-.6408	-.4743
			UUO+sh-lsg20	-.19708*	.03611	.001	-.2803	-.1138
		UUO	Control	.55149*	.03611	.000	.4682	.6348
			UUO+sh-NC	-.00603	.03611	.871	-.0893	.0772
			UUO+sh-lsg20	.35441*	.03611	.000	.2711	.4377
		UUO+sh-NC	Control	.55752*	.03611	.000	.4743	.6408
			UUO	.00603	.03611	.871	-.0772	.0893
			UUO+sh-lsg20	.36044*	.03611	.000	.2772	.4437
		UUO+sh-lsg20	Control	.19708*	.03611	.001	.1138	.2803
			UUO	-.35441*	.03611	.000	-.4377	-.2711
			UUO+sh-NC	-.36044*	.03611	.000	-.4437	-.2772
	Dunnett T3	Control	UUO	-.55149*	.03389	.001	-.7168	-.3862
			UUO+sh-NC	-.55752*	.04397	.001	-.7515	-.3636
			UUO+sh-lsg20	-.19708*	.03455	.029	-.3613	-.0328
		UUO	Control	.55149*	.03389	.001	.3862	.7168
			UUO+sh-NC	-.00603	.03761	1.000	-.2001	.1881
			UUO+sh-lsg20	.35441*	.02596	.001	.2408	.4680
		UUO+sh-NC	Control	.55752*	.04397	.001	.3636	.7515
			UUO	.00603	.03761	1.000	-.1881	.2001
			UUO+sh-lsg20	.36044*	.03820	.008	.1687	.5522
		UUO+sh-lsg20	Control	.19708*	.03455	.029	.0328	.3613
			UUO	-.35441*	.02596	.001	-.4680	-.2408
			UUO+sh-NC	-.36044*	.03820	.008	-.5522	-.1687

*. The mean difference is significant at the 0.05 level.

Fig5D

Descriptive Statistics

	N	Mean	Std. Deviation	Minimum	Maximum
TUNEL	24	15.6713	8.08538	4.82	26.20

One-Sample Kolmogorov-Smirnov Test

		TUNEL
N		24
Normal Parameters[a,b]	Mean	15.6712
	Std. Deviation	8.08538
Most Extreme Differences	Absolute	.229
	Positive	.161
	Negative	-.229
Kolmogorov-Smirnov Z		1.122
Asymp. Sig. (2-tailed)		.162

a. Test distribution is Normal.

b. Calculated from data.

Test of Homogeneity of Variances

TUNEL

Levene Statistic	df1	df2	Sig.
.539	3	20	.661

ANOVA

TUNEL

	Sum of Squares	df	Mean Square	F	Sig.
Between Groups	1453.765	3	484.588	194.530	.000
Within Groups	49.822	20	2.491		
Total	1503.586	23			

Multiple Comparisons

Dependent Variable: TUNEL

	(I) Group	(J) Group	Mean Difference (I-J)	Std. Error	Sig.	95% Confidence Interval	
						Lower Bound	Upper Bound
LSD	Control	UUO	-17.82667*	.91124	.000	-19.7275	-15.9259
		UUO+sh-NC	-17.00333*	.91124	.000	-18.9041	-15.1025
		UUO+sh-Isg20	-4.32833*	.91124	.000	-6.2291	-2.4275
	UUO	Control	17.82667*	.91124	.000	15.9259	19.7275
		UUO+sh-NC	.82333	.91124	.377	-1.0775	2.7241
		UUO+sh-Isg20	13.49833*	.91124	.000	11.5975	15.3991
	UUO+sh-NC	Control	17.00333*	.91124	.000	15.1025	18.9041
		UUO	-.82333	.91124	.377	-2.7241	1.0775
		UUO+sh-Isg20	12.67500*	.91124	.000	10.7742	14.5758
	UUO+sh-Isg20	Control	4.32833*	.91124	.000	2.4275	6.2291
		UUO	-13.49833*	.91124	.000	-15.3991	-11.5975
		UUO+sh-NC	-12.67500*	.91124	.000	-14.5758	-10.7742
Dunnett T3	Control	UUO	-17.82667*	.88743	.000	-20.7691	-14.8842
		UUO+sh-NC	-17.00333*	.83505	.000	-19.7402	-14.2665
		UUO+sh-Isg20	-4.32833*	.79473	.002	-6.9108	-1.7458
	UUO	Control	17.82667*	.88743	.000	14.8842	20.7691
		UUO+sh-NC	.82333	1.01446	.951	-2.4272	4.0739
		UUO+sh-Isg20	13.49833*	.98153	.000	10.3416	16.6551
	UUO+sh-NC	Control	17.00333*	.83505	.000	14.2665	19.7402
		UUO	-.82333	1.01446	.951	-4.0739	2.4272
		UUO+sh-Isg20	12.67500*	.93444	.000	9.6821	15.6679
	UUO+sh-Isg20	Control	4.32833*	.79473	.002	1.7458	6.9108
		UUO	-13.49833*	.98153	.000	-16.6551	-10.3416
		UUO+sh-NC	-12.67500*	.93444	.000	-15.6679	-9.6821

*. The mean difference is significant at the 0.05 level.

Fig5F

Descriptive Statistics

	N	Mean	Std. Deviation	Minimum	Maximum
BCL2	12	1.1149	.25926	.84	1.51
BAX	12	1.0522	.48419	.31	1.55

One-Sample Kolmogorov-Smirnov Test

		BCL2	BAX
N		12	12
Normal Parameters[a,b]	Mean	1.1149	1.0522
	Std. Deviation	.25926	.48419
Most Extreme Differences	Absolute	.192	.243
	Positive	.192	.166
	Negative	-.168	-.243
Kolmogorov-Smirnov Z		.666	.841
Asymp. Sig. (2-tailed)		.768	.479

a. Test distribution is Normal.

b. Calculated from data.

Test of Homogeneity of Variances

	Levene Statistic	df1	df2	Sig.
BCL2	2.401	3	8	.143
BAX	.070	3	8	.974

ANOVA

		Sum of Squares	df	Mean Square	F	Sig.
BCL2	Between Groups	.717	3	.239	83.880	.000
	Within Groups	.023	8	.003		
	Total	.739	11			
BAX	Between Groups	2.560	3	.853	355.280	.000
	Within Groups	.019	8	.002		
	Total	2.579	11			

Multiple Comparisons

Dependent Variable		(I) Group	(J) Group	Mean Difference (I-J)	Std. Error	Sig.	95% Confidence Interval	
							Lower Bound	Upper Bound
BCL2	LSD	Control	UUO	.56445*	.04357	.000	.4640	.6649
			UUO+sh-NC	.61993*	.04357	.000	.5195	.7204
			UUO+sh-Isg20	.32617*	.04357	.000	.2257	.4266
		UUO	Control	-.56445*	.04357	.000	-.6649	-.4640
			UUO+sh-NC	.05547	.04357	.239	-.0450	.1559
			UUO+sh-Isg20	-.23828*	.04357	.001	-.3388	-.1378
		UUO+sh-NC	Control	-.61993*	.04357	.000	-.7204	-.5195
			UUO	-.05547	.04357	.239	-.1559	.0450
			UUO+sh-Isg20	-.29376*	.04357	.000	-.3942	-.1933
		UUO+sh-Isg20	Control	-.32617*	.04357	.000	-.4266	-.2257
			UUO	.23828*	.04357	.001	.1378	.3388
			UUO+sh-NC	.29376*	.04357	.000	.1933	.3942
	Dunnett T3	Control	UUO	.56445*	.04117	.010	.2940	.8349
			UUO+sh-NC	.61993*	.02612	.001	.4764	.7635
			UUO+sh-Isg20	.32617*	.04073	.032	.0594	.5930
		UUO	Control	-.56445*	.04117	.010	-.8349	-.2940
			UUO+sh-NC	.05547	.04624	.779	-.1724	.2834
			UUO+sh-Isg20	-.23828	.05581	.054	-.4820	.0054
		UUO+sh-NC	Control	-.61993*	.02612	.001	-.7635	-.4764
			UUO	-.05547	.04624	.779	-.2834	.1724
			UUO+sh-Isg20	-.29376*	.04585	.023	-.5187	-.0688
BAX	LSD	Control	UUO	-1.15999*	.04001	.000	-1.2523	-1.0677
			UUO+sh-NC	-1.07883*	.04001	.000	-1.1711	-.9866
			UUO+sh-Isg20	-.60705*	.04001	.000	-.6993	-.5148
		UUO	Control	1.15999*	.04001	.000	1.0677	1.2523
			UUO+sh-NC	.08115	.04001	.077	-.0111	.1734
			UUO+sh-Isg20	.55294*	.04001	.000	.4607	.6452
		UUO+sh-NC	Control	1.07883*	.04001	.000	.9866	1.1711
			UUO	-.08115	.04001	.077	-.1734	.0111
			UUO+sh-Isg20	.47178*	.04001	.000	.3795	.5641
		UUO+sh-Isg20	Control	.60705*	.04001	.000	.5148	.6993
			UUO	-.55294*	.04001	.000	-.6452	-.4607
			UUO+sh-NC	-.47178*	.04001	.000	-.5641	-.3795
	Dunnett T3	Control	UUO	-1.15999*	.03617	.000	-1.3215	-.9985
			UUO+sh-NC	-1.07883*	.03859	.000	-1.2557	-.9020
			UUO+sh-Isg20	-.60705*	.03803	.001	-.7802	-.4339
		UUO	Control	1.15999*	.03617	.000	.9985	1.3215
			UUO+sh-NC	.08115	.04190	.427	-.1028	.2651
			UUO+sh-Isg20	.55294*	.04139	.001	.3716	.7343
		UUO+sh-NC	Control	1.07883*	.03859	.000	.9020	1.2557
			UUO	-.08115	.04190	.427	-.2651	.1028
			UUO+sh-Isg20	.47178*	.04352	.002	.2817	.6618
		UUO+sh-Isg20	Control	.60705*	.03803	.001	.4339	.7802
			UUO	-.55294*	.04139	.001	-.7343	-.3716
			UUO+sh-NC	-.47178*	.04352	.002	-.6618	-.2817

*. The mean difference is significant at the 0.05 level.
