# Supplementary material for: Targeting interferon-stimulated gene of 20 kDa protein (Isg20) inhibits ribosome biogenesis to ameliorate the progression of renal fibrosis
Source: PLoS One. 2025 Jul 7;20(7):e0322639. doi: 10.1371/journal.pone.0322639 (PMC12233288; doi:10.1371/journal.pone.0322639)
Supplement: S3 File — (DOCX) [file pone.0322639.s003.docx]

**S3 Table. Expression of RiboSis-related differentially expressed genes with the top 10 log_2_|FC| values from GSE42303 and GSE121190**

| Gene | Data | \|logFC\| | Reg | P | Adjp |
| --- | --- | --- | --- | --- | --- |
| *Rps19* | GSE121190 | 1.968134 | Up | 6.83E-06 | 0.001233 |
| *Rexo4* | GSE121190 | 1.908265 | Up | 0.00372 | 0.038719 |
| *Isg20* | GSE121190 | 1.655948 | Up | 0.00339 | 0.036779 |
| *Rpp25* | GSE121190 | 1.591007 | Up | 0.0289 | 0.130141 |
| *Rps5* | GSE121190 | 1.557729 | Up | 1.42E-05 | 0.001714 |
| *Rps9* | GSE121190 | 1.527057 | Up | 0.000791 | 0.01577 |
| *Rpl27* | GSE121190 | 1.298194 | Up | 5.80E-06 | 0.001185 |
| *Rps14* | GSE121190 | 1.179743 | Up | 0.0149 | 0.088044 |
| *Gtf3c4* | GSE121190 | 1.0937892 | Up | 0.000618 | 0.0138666 |
| *Gtf3c6* | GSE121190 | -1.81386 | Down | 1.33E-05 | 0.00165 |
| *Rps25* | GSE42303 | 1.165258 | Up | 0.00413 | 0.028941 |
| *Rps9* | GSE42303 | 0.84496 | Up | 0.000608 | 0.010359 |
| *Rpl35* | GSE42303 | 0.800326 | Up | 0.000771 | 0.011843 |
| *Rps19* | GSE42303 | 0.79069 | Up | 0.000863 | 0.012687 |
| *Isg20* | GSE42303 | 0.774861 | Up | 0.00158 | 0.017471 |
| *Rps7* | GSE42303 | 0.704321 | Up | 0.00108 | 0.014309 |
| *Rpsa* | GSE42303 | 0.696821 | Up | 0.00291 | 0.023964 |
| *Rps10* | GSE42303 | 0.674354 | Up | 0.00556 | 0.03396 |
| *Utp6* | GSE42303 | -1.10315 | Down | 0.000875 | 0.012791 |
| *Mtor* | GSE42303 | -1.21814 | Down | 0.00162 | 0.01769 |
